# Beyond Synovitis: Molecular and Neuroimmune Mechanisms Informing Target-Joint Attribution and Pain Phenotyping for Arthroplasty in Inflammatory Arthritis

**DOI:** 10.3390/ijms27198551

**Published:** 2026-09-24

**Authors:** Michal Pruc, Andrzej Bielski, Robert Weglowski, Jaroslaw Pecold, Lukasz Szarpak, Iwona Jannasz, Monika Olczak-Pruc, Maciej Lewicki, Maciej Maslyk, Mahdi Al-Jeabory

**Affiliations:** 1 Institute of Medical Science, The John Paul II Catholic University of Lublin, 20-708 Lublin, Poland; 2 Clinical Department of Rheumatology, Provincial Integrated Hospital in Plock, 09-400 Plock, Poland; 3 Collegium Medicum, The Mazovian Academy in Plock, 09-402 Plock, Poland; 4 Clinical Department of Trauma and Orthopedic Surgery, The John Paul II Catholic University of Lublin, 21-300 Radzyn Podlaski, Poland; 5 Department of Trauma and Orthopedic Surgery, Ruda Slaska City Hospital, 41-793 Ruda Slaska, Poland; 6 Henry JN Taub Department of Emergency Medicine, Baylor College of Medicine, Houston, TX 77030, USA; 7 Department of Geriatrics, National Institute of Geriatrics, Rheumatology and Rehabilitation, 02-637 Warsaw, Poland; 8 Rheumatology Unit, ViaMed Ursus Medical Center, 02-495 Warsaw, Poland; 9 Department of Rheumatology, Silesian Center for Rheumatology, 43-450 Ustron, Poland; 10 Institute of Biological Science, The John Paul II Catholic University of Lublin, 20-950 Lublin, Poland; 11 Department of Trauma and Orthopedic Surgery, Silesian Center for Rheumatology, 43-450 Ustron, Poland; 12 Clinical Department of Orthopedics, Faculty of Medical Sciences in Zabrze, Medical University of Silesia, 41-800 Katowice, Poland

**Keywords:** inflammatory arthritis, rheumatoid arthritis, arthroplasty, nociplastic pain, central sensitisation, distributed pain amplification, pain phenotyping, neuroimmune signalling, cytokines, nerve growth factor, molecular biomarkers

## Abstract

Arthroplasty can provide substantial pain and functional improvement in inflammatory arthritis, but structural indications alone do not determine how much of a patient’s overall symptom burden will respond to surgery. Persistent or widespread pain may reflect active inflammation, irreversible joint damage, neuropathic or extra-articular generators, and distributed pain amplification, which we use as a descriptive term for pain extending beyond the target joint and influenced by broader sensory, symptomatic, and contextual processes. This critical structured narrative review integrates direct inflammatory-arthritis arthroplasty evidence, disease-specific pain-phenotyping studies, indirect osteoarthritis perioperative data, and molecular and mechanistic literature. We link cytokine/prostaglandin signalling, neurotrophin- and ion-channel-mediated nociceptor sensitisation, immunosensory signalling, and spinal neuron–glia plasticity to peripheral and central nociceptive amplification. We then propose the Target-Joint Attribution and Pain-Amplification Framework, comprising four provisional clinical phenotypes (A–D): structural-concordant, inflammation-dominant, mixed target-joint/distributed pain-amplification, and distributed pain-dominant. Questionnaire or sensory-test thresholds do not establish mechanisms or surgical eligibility. The framework translates these molecular and neuroimmune mechanisms into testable hypotheses for perioperative phenotyping, biomarker validation, and interpretation of postoperative pain trajectories. Direct phenotype-specific evidence in inflammatory-arthritis arthroplasty remains sparse; prospective validation integrating joint-specific outcomes with molecular and sensory measures is required.

## 1. Introduction: The Attribution Problem

Consider a hypothetical 58-year-old woman with rheumatoid arthritis who has a destroyed, varus knee. Her C-reactive protein is low, no swollen joints are evident, and power Doppler ultrasonography shows little synovial signal. Loading the knee reproduces sharp medial pain; she cannot climb stairs or rise from a low chair. She also reports lumbar pain, aching in both shoulders and the opposite leg, unrefreshing sleep, fatigue, and an expectation that replacement will remove all pain. The radiograph supports arthroplasty. The consultation has not yet established which symptoms are likely to respond.

Technical success, relief of pain in the operated joint, reduction in global pain, functional recovery, satisfaction, and fulfilment of expectations are related but distinct outcomes. Rheumatoid arthritis cohorts show substantial benefit after hip or knee replacement despite worse baseline status and, in some studies, worse absolute postoperative pain or function than in osteoarthritis [1,2,3]. Contemporary pooled data show unfavourable long-term pain outcomes in approximately 12–15% of patients from 6 months to 2 years after total knee replacement and in at least 14% at 6–12 months after total hip replacement, although estimates vary by definition and follow-up while confidence is limited by heterogeneity and risk of bias [4]. The appropriate response is a more exact forecast of what the operation can address.

Rheumatology research has concentrated on residual pain, discordant disease activity, fibromyalgia traits, sensory hypersensitivity, and increasingly mechanism-informed approaches to pain management in inflammatory arthritis [5]. Arthroplasty research has concentrated on persistent postoperative pain, chiefly in osteoarthritis. These strands have rarely been integrated into an arthroplasty-specific assessment framework centred on target-joint attribution in inflammatory arthritis. The central question is not simply whether features consistent with a nociplastic component are present. It is whether the target joint remains a dominant, surgically remediable generator within a mixed pain state [1,2,3,4,5].

The novelty of this review lies in integrating four evidence domains that have largely been considered separately: molecular and neuroimmune pain mechanisms in inflammatory arthritis, disease-specific pain phenotyping, direct arthroplasty evidence in inflammatory arthritis, and indirect perioperative evidence from osteoarthritis. We use this synthesis to address a specific clinical problem: whether the joint considered for replacement remains a dominant, surgically remediable pain generator when structural, inflammatory, neuropathic, and distributed pain mechanisms coexist. On this basis, we propose the Target-Joint Attribution and Pain-Amplification Framework, which separates target-joint attribution, distributed pain amplification, and the preparation/recovery context. The framework is hypothesis-generating and is not intended as a diagnostic score or surgical exclusion rule.

## 2. Methods: Critical Structured Narrative Review and Conceptual Synthesis

This critical, structured narrative review and conceptual synthesis were undertaken to examine the molecular and neuroimmune mechanisms linking inflammatory joint pathology to persistent and distributed pain amplification and to translate these mechanisms into perioperative pain phenotyping and target-joint attribution in adults with inflammatory arthritis being considered for arthroplasty. The review aimed to: (1) synthesise molecular and neuroimmune pathways connecting synovitis and tissue injury with peripheral sensitisation, spinal neuroimmune plasticity, and persistent pain amplification; (2) distinguish mechanistic plausibility from clinically validated molecular biomarkers; (3) characterise pain-related phenotypes and clinical findings that support or weaken target-joint attribution; (4) examine direct and indirect evidence linking preoperative pain-related features with arthroplasty outcomes; and (5) develop a hypothesis-generating translational framework for assessment, preparation, counselling, and outcome interpretation.

The conduct and reporting of this review were informed by the principles represented in the Scale for the Assessment of Narrative Review Articles (SANRA), particularly the articulation of the review’s importance and objectives, transparency of literature searching, appropriate referencing, scientific reasoning, and presentation of relevant endpoint data [6]. It was not prospectively registered and was not designed to meet the methodological requirements of a systematic review, scoping review, or meta-analysis. The searches were therefore structured and iterative rather than exhaustive, and no pooled effect estimates were generated.

Targeted literature searches were conducted in PubMed, Embase, Scopus, Web of Science, and the Cochrane Library from each database’s inception to 16 July 2026. A focused update check of PubMed and relevant professional-society guidance was performed on 10 August 2026 to capture newly published material after the main search. Additional targeted searches prompted by reviewer comments were undertaken during revision through 16 September 2026. Search concepts combined controlled vocabulary, where available, and free-text terms relating to rheumatoid arthritis, psoriatic arthritis, spondyloarthritis, inflammatory arthritis, arthroplasty or joint replacement, pain distribution and amplification, nociplastic and neuropathic pain, fibromyalgia traits, sensory testing, persistent postoperative pain, function, satisfaction, expectations, sleep, psychological factors, opioid exposure, cytokines, neurotrophins, nociceptor ion channels, neuroimmune signalling, glial activation, and autoantibody or immune-complex signalling.

Searches were organised around four complementary evidence streams: molecular and neuroimmune evidence relevant to pain generation and amplification in inflammatory arthritis; disease-specific pain-phenotyping studies in inflammatory arthritis; direct evidence from arthroplasty populations with inflammatory arthritis; and indirect perioperative evidence from osteoarthritis and mixed arthroplasty populations. Mechanistic evidence was used to define biological plausibility and candidate translational pathways, whereas clinical and perioperative evidence was used to examine whether these mechanisms have validated implications for target-joint attribution, phenotyping, prognosis, or postoperative outcome interpretation. Search terms and emphasis were refined iteratively as clinically relevant concepts and evidence gaps emerged. Searches were supplemented by backward citation searching of relevant reviews and included studies and by forward citation searching of selected index publications. Only English-language publications were considered.

Adult human and translational studies were prioritised within each evidence stream when available. For molecular and neuroimmune questions, human mechanistic data were used preferentially, supplemented by preclinical cellular and animal studies when the relevant pathways could not be examined directly in humans. Such preclinical evidence was used to establish biological plausibility rather than clinical effect size, perioperative thresholds, or surgical eligibility. For perioperative translation, priority was given to studies combining inflammatory arthritis, arthroplasty, preoperative pain-related assessment, and postoperative outcomes. Where direct perioperative evidence was unavailable or insufficient, disease-specific inflammatory-arthritis studies were considered, followed by arthroplasty studies in osteoarthritis or mixed populations. Randomised and observational studies, systematic reviews, meta-analyses, and clinical guidance were eligible. When multiple publications described overlapping cohorts, preference was given to the report with the most relevant outcomes, follow-up duration, or adjusted analysis.

Identification and consideration of the literature were primarily undertaken by one reviewer. A second reviewer checked studies considered in the synthesis in the direct inflammatory-arthritis arthroplasty evidence stream, publications supporting numerical estimates or major framework components, and records for which eligibility or interpretation was uncertain. Key clinical studies were charted in a structured evidence matrix covering population, study design, procedure, pain-related assessment, outcome definition, follow-up, effect estimates, covariate adjustment, relevance to inflammatory-arthritis arthroplasty, and principal limitations. Numerical estimates presented in the manuscript were checked against the corresponding original publications by a second author.

Formal study-level risk-of-bias scoring and certainty grading were not undertaken because the purpose of the review was critical narrative synthesis and framework development rather than estimation of a pooled treatment effect or formal assessment of intervention efficacy. Studies supporting major clinical conclusions, numerical estimates, or framework components were nevertheless critically appraised with respect to population relevance, validity of exposure and outcome measurement, separation of target-joint and global outcomes, temporal sequence, adjustment for baseline pain and confounding, completeness of follow-up, statistical precision, common-method variance, and directness to inflammatory-arthritis arthroplasty.

Findings were synthesised narratively and described according to their evidential role as direct inflammatory-arthritis arthroplasty evidence, disease-specific inflammatory-arthritis evidence, indirect arthroplasty evidence, mechanistic evidence, or expert inference. Observational associations were not interpreted as proof of causality, mechanism, or treatment modifiability. Particular caution was applied when extrapolating evidence from osteoarthritis, total knee arthroplasty, or mixed arthroplasty populations to patients with inflammatory arthritis.

Candidate framework components were developed through iterative conceptual synthesis. Components were retained when they were clinically observable, conceptually distinguishable, relevant to perioperative decision-making, and supported either by empirical evidence or by an explicitly identified mechanistic rationale. The framework was reviewed iteratively by the author group to reduce conceptual overlap, consider competing explanations, and preserve the distinction between pain-source attribution, prognostic modifiers, and recovery context. It was not derived from statistical clustering, developed through formal consensus methods, or validated as a diagnostic or prognostic model. It should therefore be interpreted as a hypothesis-generating clinical reasoning framework rather than a decision rule or surgical eligibility instrument.

## 3. Mechanistic Terminology and Conceptual Boundaries

Pain is an unpleasant sensory and emotional experience associated with, or resembling that associated with, actual or potential tissue damage [7]. Inflammatory nociceptive pain arises when inflammatory mediators activate or sensitise peripheral nociceptors. Structural nociceptive pain arises from damaged, deformed, unstable, or abnormally loaded tissues. In this review, “structural nociceptive pain” is used pragmatically to describe nociception arising from damaged or mechanically abnormal joint tissues; it is not proposed as a separate formal International Association for the Study of Pain (IASP) mechanistic category. Inflammatory and structural nociceptive pain may coexist. Peripheral sensitisation describes increased nociceptor responsiveness near inflamed or injured tissue, producing local tenderness, movement-evoked pain, and primary hyperalgesia.

Central sensitisation is a neurophysiological process in which nociceptive neurons within the central nervous system show increased responsiveness to normal or subthreshold input [8,9]. Nociplastic pain is a clinical mechanistic descriptor for pain arising from altered nociception when ongoing tissue damage—or a lesion or disease of the somatosensory system—activating the peripheral nociceptors does not fully explain the presentation [10,11]. These constructs overlap but are not interchangeable. A nociplastic component can coexist with active synovitis, severe joint destruction, radiculopathy, or peripheral neuropathy.

Neuropathic pain requires a somatosensory lesion or disease, graded from neuroanatomical history, sensory signs, and confirmatory testing where appropriate [12]. Neuropathic-like symptoms are questionnaire-defined qualities; they do not establish a lesion. Fibromyalgia combines widespread pain with associated symptoms; survey criteria quantify its traits but not the mechanism at a particular joint [13,14]. Chronic postsurgical pain develops or increases in intensity after surgery, persists beyond the healing process (defined as at least 3 months after surgery), is localised to the surgical field, the territory of a nerve within that field, or an anatomically plausible referral area, and is not better explained by infection, malignancy, mechanical failure, or another pre-existing pain condition [15].

The Central Sensitization Inventory (CSI) is a broad symptom inventory covering sleep, fatigue, cognitive, autonomic, and emotional items [16,17]. It does not diagnose central sensitisation. Meta-analyses show closer alignment with self-reported symptom and psychological constructs than experimental nociceptive measures; CSI scores do not reliably identify impaired conditioned pain modulation [18,19]. Recent interdisciplinary guidance further cautions that central sensitisation should not be inferred from symptoms or questionnaires alone; in clinical contexts, the distinction between a neurophysiological phenomenon and indirect clinical features should be made explicit [20]. PainDETECT screens for neuropathic-like descriptors; it does not diagnose neuropathic or nociplastic pain [21].

Quantitative sensory testing (QST) samples pressure-pain thresholds, temporal summation, and conditioned pain modulation [22,23]. The results depend on the protocol, body site, medication, sleep, expectation, and examiner technique. A 2025 individual-participant-data meta-analysis found no consistent person-level prediction of pain or disability across lower-limb osteoarthritis treatments [24]. No QST measure is a gold standard. Psychological distress can modify pain and recovery without diminishing symptom credibility. Mixed states require convergent evidence and reassessment (Table 1).

## 4. Clinical Context for Molecular and Neuroimmune Pain Amplification in Inflammatory Arthritis

Inflammatory arthritis introduces pain and recovery risks not captured by the usual osteoarthritis phenotype. In rheumatoid arthritis, synovitis may sustain peripheral input, while earlier inflammation leaves cartilage loss, erosions, deformity, ligamentous failure, and secondary osteoarthritis. Pain may therefore persist despite systemic control. In rheumatoid arthritis, a normal C-reactive protein concentration, absent or minimal power Doppler synovial signal, or apparent clinical remission reduces the likelihood of active synovitis at the assessed site, but this does not exclude a structural, extra-synovial, or mechanically mediated source of pain. Imaging should be interpreted alongside examination, anatomical concordance, and functional testing rather than used alone to adjudicate the source of pain [28,29].

Composite disease-activity scores can also be distorted by pain mechanisms. Tender-joint counts and patient global assessment may rise with multisite pain, poor sleep, fatigue, mood disturbance, or fibromyalgia traits while swollen-joint counts and inflammatory markers remain low [30,31]. Selected rheumatoid arthritis QST cohorts report lower pressure-pain thresholds, enhanced responses, or altered inhibition [32,33,34,35]. The relationship with inflammation is not uniform. In clinically active disease, inflammatory markers may explain more pain variance than QST indices; other cohorts show sensory hypersensitivity associated with pain beyond objective inflammatory activity [36]. A 2025 exploratory study examining rheumatoid arthritis, psoriatic arthritis, and axial spondyloarthritis within a four-disease cohort found somatosensory abnormalities and pathological allodynia that were not associated with disease activity or inflammatory markers; however, only 20 patients per disease group were assessed at the hands [37].

Psoriatic arthritis adds enthesitis, dactylitis, axial symptoms, obesity, skin disease, and degenerative comorbidity. Cross-sectional CSI studies associate higher scores with disability and composite disease activity, but they mainly quantify symptom burden [38,39]. In a 2026 single-centre cross-sectional study of 228 patients with psoriatic arthritis without fibromyalgia, 35.1% met a CSI threshold of 40; depression, poor sleep, and painDETECT scores were independent correlates, and the design did not establish mechanism or causality [40]. The clinically relevant point is discordance, not a prevalence label.

Axial spondyloarthritis combines inflammatory back pain, structural spinal change, hip disease, enthesitis, and widespread pain. Fibromyalgia is over-represented, and CSI scores are strongly shaped by fatigue, anxiety, catastrophising, and illness perception [41,42]. QST differences occur at the group level, but impaired conditioned pain modulation is not a consistent finding [43,44]. Widespread pain can materially influence patient-reported global health and disease-activity indices [45,46].

Finally, long-standing inflammatory disease is often accompanied by substantial fatigue and reduced physical capacity, while multimorbidity and damage at multiple musculoskeletal sites can further complicate attribution and rehabilitation. Fatigue itself has heterogeneous determinants and consequences across rheumatic and musculoskeletal diseases [47]. Perioperative immunomodulatory management introduces separate infection and flare considerations. These factors alter attribution and recovery reserves independently of any pain label. These disease-specific clinical features therefore provide the context in which molecular and neuroimmune pain mechanisms must be interpreted; they should not be treated as independent mechanistic labels or as stand-alone determinants of surgical eligibility.

## 5. Molecular and Neuroimmune Mechanisms Linking Synovitis to Persistent and Distributed Pain Amplification

The mechanistic model is dynamic (Figure 1). In this review, distributed pain amplification is used as a descriptive umbrella term for convergent evidence that pain extends beyond the target joint and is influenced by broader sensory, symptomatic, and contextual processes. It is not a formal IASP diagnosis and does not, by itself, establish central sensitisation or nociplastic pain. Nociplastic pain is used more narrowly when the clinical presentation meets accepted mechanism-informed criteria and is not fully explained by nociceptive or neuropathic mechanisms alone [11]. Synovitis and tissue injury increase afferent nociceptive traffic. Repeated input can produce local sensitisation, spinal facilitation, enhanced temporal summation, and less effective descending inhibition. Sleep disruption and fatigue lower tolerance for nociceptive input; reduced activity then worsens strength, gait efficiency, confidence, and metabolic health. Fear, social adversity, and repeated treatment failure can reinforce vigilance and avoidance. None of these processes implies that the target joint is incidental.

We hypothesise that, in inflammatory arthritis, sustained joint-derived nociceptive input from active inflammation and/or irreversible structural damage increases peripheral nociceptor gain and, with repeated or prolonged exposure, can recruit spinal and supraspinal neuroimmune plasticity that broadens pain beyond the target joint. Within this model, arthroplasty may reduce a dominant peripheral pain generator but does not necessarily reverse established distributed pain amplification. Postoperative pain therefore reflects the balance between removal of joint-derived nociceptive input, surgery-induced nociception or peripheral nerve injury, and the persistence or reversibility of central neuroimmune changes. This hypothesis predicts that patients with strong target-joint attribution and limited distributed amplification should, on average, experience greater joint-specific pain relief than patients in whom distributed amplification is prominent, even when structural joint disease is similarly advanced. This is a hypothesis-generating model rather than a validated causal pathway or prognostic rule.

### 5.1. Cytokine and Prostaglandin Signalling

Inflammatory arthritis arises from a multicellular synovial microenvironment rather than from individual cytokines acting in isolation. Fibroblast-like synoviocytes, resident and infiltrating synovial macrophages, adaptive immune cells, vascular cells, and cells involved in cartilage and bone remodelling form interacting networks that sustain inflammation and structural damage. In rheumatoid arthritis, activated fibroblast-like synoviocytes acquire persistent pro-inflammatory and tissue-invasive phenotypes, producing cytokines, chemokines, matrix-degrading enzymes, and mediators that promote immune-cell recruitment and osteoclast activation. Synovial macrophages are an important source of TNF and other inflammatory mediators, while adaptive immune cells further shape fibroblast, macrophage, and osteoclast activity. Single-cell and functional studies additionally indicate that synovial fibroblasts and macrophages are heterogeneous, with distinct subsets contributing preferentially to inflammation, tissue destruction, or maintenance of joint homeostasis [48,49,50]. These cellular interactions provide the biological context in which cytokine, prostaglandin, neurotrophin, and nociceptor-sensitising pathways operate.

At the molecular level, inflammatory joint pain begins with bidirectional signalling between immune and stromal cells and nociceptors rather than with tissue damage alone. Tumour necrosis factor alpha (TNF-α), interleukin-1 beta (IL-1β), interleukin-6 (IL-6), and interleukin-17 (IL-17) can act directly on subsets of sensory neurons that express cognate receptors, changing membrane excitability and second-messenger signalling, while inflammatory lipid mediators, such as prostaglandin E2 (PGE2), further lower activation thresholds [51,52]. These pathways converge on transduction machinery including transient receptor potential channels and voltage-gated sodium channels, increasing responses to mechanical, thermal, and chemical stimuli. This provides a molecular basis for peripheral sensitisation during active synovitis, but it also means that pain intensity need not track a single systemic inflammatory marker. Importantly, peripheral pro-inflammatory cytokine gene expression may remain limited even in patients with prominent pain-amplification features. In a recent exploratory study of rheumatoid arthritis, PBMC gene-expression analysis across CSI-defined symptom-severity groups showed IL-1β expression comparable with healthy controls, whereas TNF-α expression was increased only in patients with severe/extreme CSI scores [53]. These findings suggest that substantial central-sensitisation-related symptom burden can coexist with relatively low peripheral pro-inflammatory cytokine gene expression. However, the CSI-based grouping does not establish neurophysiological central sensitisation, and PBMC transcription should not be interpreted as a surrogate for cytokine activity within synovial tissue, dorsal-root ganglia, or the central nervous system. Cytokine blockade may therefore reduce pain partly through suppression of inflammation and partly through direct or indirect effects on neuronal sensitisation; the relative contribution is context-dependent and cannot be inferred from response to a single drug.

### 5.2. Neurotrophic Signalling: NGF–TrkA, GDNF Family Ligands, and Nociceptor Ion-Channel Sensitisation

Neurotrophin signalling provides a second bridge between tissue pathology and nociceptor plasticity. Nerve growth factor (NGF) released within inflamed or mechanically stressed tissues binds tropomyosin receptor kinase A (TrkA) on nociceptors and can enhance the function or trafficking of transient receptor potential vanilloid 1 (TRPV1) and related transduction pathways, all while promoting neuropeptide release and longer-term changes in the sensory-neuron phenotype [54]. In bone and joint disorders, NGF biology also intersects with tissue remodelling and angiogenesis, so a molecular pathway that amplifies pain is not necessarily a safe or selective perioperative therapeutic target. For arthroplasty assessment, the translational point is narrower: sustained neurotrophin-dependent sensitisation can increase the gain of a genuine peripheral generator without proving that the pain is predominantly nociplastic or centrally maintained. A destroyed, mechanically concordant joint may therefore remain surgically remediable, even when the nociceptive system has become sensitised.

Beyond NGF, members of the glial cell line-derived neurotrophic factor (GDNF) family provide additional routes through which inflammatory and tissue-derived signals may modify nociceptor gain. Artemin (ARTN) signals preferentially through GDNF family receptor alpha 3 (GFRα3)–rearranged during transfection receptor tyrosine kinase (RET), whereas neurturin (NRTN) predominantly engages GDNF family receptor alpha 2 (GFRα2)–RET; persephin (PSPN) is the corresponding ligand for GDNF family receptor alpha 4 (GFRα4), although its role in peripheral nociception is much less clearly established [55]. Experimental studies show that ARTN and NRTN can activate or sensitise bone afferent neurons, and that sequestration of these ligands can reduce inflammatory bone-pain behaviour [56]. ARTN/GFRα3 signalling has also been implicated in osteoarthritis-related joint and bone hypersensitivity, with experimental ARTN exposure increasing mechanical and thermal sensitivity and ARTN sequestration reversing hypersensitivity in preclinical models [57]. PGE2 and ARTN should therefore be viewed as potentially convergent rather than necessarily serial mediators. PGE2 signalling through prostaglandin E receptors can enhance nociceptor excitability through pathways involving TRPV1 and voltage-gated sodium channels, whereas ARTN–GFRα3–RET signalling can increase the responsiveness of overlapping nociceptive transduction machinery. Recent synthesis specifically describes convergence of PGE2 and ARTN signalling at TRPV1-dependent peripheral sensitisation [58]. This provides a plausible route for amplification of inflammatory or mechanically driven pain, but a direct causal PGE2–ARTN axis has not been validated in inflammatory-arthritis arthroplasty populations. A complementary counter-regulatory pathway, TAFA chemokine-like family member 4 (TAFA4)-associated signalling, is discussed below; experimental data indicate antihypersensitivity actions involving low-density lipoprotein receptor-related protein (LRP)-dependent spinal modulation and formyl peptide receptor 1 (FPR1)/neurexin-associated neuroimmune signalling [59,60].

### 5.3. Immune-Complex/Fc Gamma Receptor Signalling

Inflammatory arthritis also illustrates immune-to-neuron signalling that can be partly uncoupled from overt synovitis. In experimental systems, cartilage-binding antibodies directed against type II collagen or cartilage oligomeric matrix protein can form local immune complexes and activate primary afferents through neuronal Fc gamma-receptor mechanisms, producing mechanical hypersensitivity without parallel histological inflammation [61]. Complementary work has shown functional Fc gamma receptor I (FcγRI) expression in joint sensory neurons and attenuation of arthritis pain after neuronal FcγRI disruption without equivalent reduction in joint inflammation [62]. These findings are preclinical and should not be extrapolated into a clinical biomarker or surgical selection test, but they are important conceptually: immune activity can influence pain through direct immunosensory signalling, helping to explain why pain and conventional measures of inflammatory activity may diverge. This is particularly relevant to a framework that separates current inflammatory control from target-joint attribution.

### 5.4. Spinal Neuron–Glia Plasticity

Persistent afferent input can be amplified within the spinal cord through activity-dependent and neuroimmune plasticity. Arthritis models support roles for glutamatergic α-amino-3-hydroxy-5-methyl-4-isoxazolepropionic acid (AMPA) receptor and N-methyl-D-aspartate (NMDA) receptor signalling, neuropeptides, prostaglandins, and spinal cytokines together with activation of microglia and astrocytes [63]. Microglia–neuron signalling can modify inhibitory control. In other pain models, adenosine triphosphate (ATP)-driven microglial brain-derived neurotrophic factor (BDNF) acting through tropomyosin receptor kinase B (TrkB) shifts the neuronal chloride gradient [64]; complementary experimental work shows that BDNF-TrkB signalling can down-regulate potassium-chloride cotransporter 2 (KCC2) and impair neuronal chloride extrusion [65]. Arthritis-specific evidence also implicates purinergic and p38 mitogen-activated protein kinase (p38 MAPK)-linked glial signalling, cathepsin S/C-X3-C motif chemokine receptor 1 (CX3CR1), high-mobility group box 1 (HMGB1)/Toll-like receptor 4 (TLR4), and nuclear factor kappa B (NF-κB)-related pathways [63]. These data provide biological plausibility for remote hypersensitivity, enhanced temporal summation, and persistence of pain after the peripheral inflammatory state has improved. However, much of the detailed molecular evidence is preclinical, and we identified no cytokine, neurotrophin, autoantibody, or glial marker that has been validated as a perioperative classifier of arthroplasty benefit in inflammatory arthritis. A further translational obstacle is biological compartmentalisation. For a neuroimmune pathway marker to become clinically useful, it should ideally be reproducibly measurable in peripheral blood or another clinically accessible biofluid and its measured level should meaningfully reflect activity within the relevant joint or neural compartment. This requirement is especially challenging for predominantly central neuroimmune processes, because molecular changes within the dorsal-root ganglia, spinal cord, or cerebrospinal fluid may not generate a detectable or specific peripheral signal. In a human chronic-pain study using paired serum and cerebrospinal-fluid sampling, central neuroimmune activation was observed in the absence of systemic inflammation, illustrating that central and peripheral inflammatory compartments can diverge [66]. Accordingly, analytical detectability in an accessible biofluid and demonstrated biological correspondence between the sampled and disease-relevant compartments should be treated as distinct prerequisites for biomarker validation. Molecular mechanisms should therefore sharpen mechanistic hypotheses and biomarker research, not replace clinical attribution.

An additional potentially counter-regulatory pathway is TAFA4 signalling. TAFA4, a chemokine-like protein expressed by subsets of sensory neurons, has been linked to formyl peptide receptor 1 (FPR1)- and neurexin-associated signalling, while experimental antihypersensitivity also requires low-density lipoprotein receptor-related proteins (LRPs) [59,60]. In animal models, TAFA4 reverses inflammatory, postoperative, and nerve-injury-induced mechanical hypersensitivity and can restore spinal neuronal excitability toward baseline [59]. These observations position TAFA4 differently from PGE2 and ARTN; rather than simply increasing peripheral nociceptor gain, TAFA4 may represent an endogenous pathway that limits or reverses pain amplification. Direct interaction between TAFA4 and PGE2 signalling has not been established, and the evidence remains predominantly preclinical.

### 5.5. Emerging Human Molecular Signatures and Type 1 Interferon Signalling

Exploratory human data provide a preliminary translational bridge. In a 2026 cross-sectional study of 59 patients with rheumatoid arthritis, CSI-defined symptom-severity groups were examined alongside peripheral blood mononuclear cell (PBMC) gene-expression profiles. Gene-expression analysis showed alterations in pathways related to metabolism, oxidative defence, apoptosis, tissue destruction, and hypernociception, while pro-inflammatory cytokine expression remained comparatively limited; IL-1β expression was similar to that in healthy controls and TNF-α was increased only in the severe/extreme CSI subgroup [53]. However, the peripheral sampling compartment, cross-sectional design, and questionnaire-defined grouping preclude interpreting these findings as a molecular signature of central sensitisation or as a perioperative biomarker.

Recent translational work adds a distinct type 1 interferon (IFN) pathway. In autoantibody-induced arthritis, persistent interferon alpha/beta (IFN-α/β) signalling through neuronal interferon alpha/beta receptor (IFNAR) engaged MAP kinase-interacting serine/threonine-protein kinases 1 and 2 (MNK1/MNK2)–eukaryotic translation initiation factor 4E (eIF4E) signalling and sustained joint-afferent hyperexcitability before, during, and after overt inflammation; human dorsal-root-ganglion samples from individuals with painful rheumatoid arthritis showed increased type 1 IFN expression and pathway activation [67]. These findings strengthen the concept that immunosensory signalling can become partly uncoupled from conventional measures of synovitis, but they do not establish a circulating biomarker, perioperative threshold, or treatment strategy for arthroplasty selection.

### 5.6. Osteoarthritis-Related and Postsurgical Mechanisms of Persistent Pain After Arthroplasty

Because long-standing inflammatory arthritis may coexist with secondary osteoarthritis, some patients enter arthroplasty with both inflammatory-arthritis and osteoarthritis-associated nociceptive drivers. In osteoarthritis, chronic synovitis and inflammation, together with persistent peripheral nociceptive input, can promote peripheral and central sensitisation before surgery; preoperative central sensitisation has been associated with persistent pain after knee and hip arthroplasty [68]. Arthroplasty removes diseased joint tissue but also produces acute tissue injury and, in some cases, peripheral nerve injury. Surgical trauma can release adenosine triphosphate and damage-associated molecular patterns and increase local tumour necrosis factor alpha, interleukin-1 beta, chemokines, and other inflammatory mediators, lowering nociceptor thresholds and sustaining peripheral sensitisation. Persistent afferent activity and nerve injury may then reinforce dorsal-horn excitability, N-methyl-D-aspartate receptor signalling, microglial and astrocytic activation, and neuroimmune chemokine and Toll-like-receptor pathways, providing a biological route from acute postoperative pain to chronic postsurgical pain [69]. These mechanisms overlap with the cytokine, ion-channel, and neuron–glia pathways described above; they are not unique to osteoarthritis and do not imply that pain after arthroplasty is centrally maintained in every patient. Persistent pain after osteoarthritis arthroplasty is therefore best viewed as potentially arising from the interaction of pre-existing sensitisation, surgery-induced inflammation or nerve injury, and ongoing central neuroimmune plasticity rather than from a single molecular pathway [68,69].

Arthroplasty also initiates a local wound-healing response involving macrophages, fibroblasts, vascular cells, and recruited immune cells. In uncomplicated healing, this response resolves as tissue repair progresses; however, excessive or persistent signalling can promote fibroblast-to-myofibroblast differentiation, extracellular matrix deposition, and pathological fibrosis. Transforming growth factor beta (TGF-β) is a central profibrotic mediator acting together with inflammatory cytokines and other tissue-repair signals to sustain myofibroblast activation. This process is particularly relevant to postoperative arthrofibrosis, in which persistent fibroblast and myofibroblast activity and excessive collagen deposition can contribute to stiffness, pain, and impaired function after arthroplasty [70]. These postoperative fibrotic mechanisms should be distinguished from the pre-existing autoimmune synovial pathology of inflammatory arthritis, although inflammatory and wound-healing pathways may interact in individual patients.

### 5.7. Integrated Mechanistic Model and Translational Relevance

For the Target-Joint Attribution and Pain-Amplification Framework, these pathways are best interpreted as layered modifiers. Synovial cytokines and PGE2 help explain inflammation-dominant states; NGF–TrkA, ARTN/GFRα3–RET, NRTN/GFRα2–RET, and ion-channel sensitisation can amplify structurally concordant peripheral pain; immune-complex/Fcγ-receptor signalling and type 1 interferon–IFNAR–MNK1/MNK2–eIF4E signalling demonstrate that immunological pain can be partly uncoupled from routine inflammatory markers; TAFA4-associated signalling provides a candidate counter-regulatory mechanism; and spinal neuron–glia plasticity provides a plausible substrate for distributed amplification [51,52,54,55,56,57,58,59,60,61,62,63,64,65,66,67]. None map one-to-one onto the phenotypes A–D. The same patient may have more than one active pathway, and molecular signatures may change with treatment, disease stage, and removal of the target joint. A clinically useful biomarker would therefore need to add information beyond structural severity, objective inflammation, pain distribution, and baseline symptom burden and, crucially, predict joint-specific benefit rather than merely correlate global pain. These molecular pathways operate within a multicellular synovial and periarticular network in which fibroblasts, macrophages, adaptive immune cells, vascular cells, nociceptors, and bone-remodelling cells can mutually reinforce or restrain inflammation, tissue damage, repair, and nociceptive signalling [48,49,50].

Inflammatory and structural joint pathology can sustain peripheral input and distributed amplification. The molecular callouts show representative mechanisms—cytokine and prostaglandin signalling, NGF–TrkA and ion-channel sensitisation, glutamatergic transmission, and neuron–glia signalling—rather than an exhaustive pathway map or validated perioperative biomarkers [51,52,54,61,62,63,64,65]. Additional candidate pathways, including GDNF family ligand signalling and TAFA4-associated modulation, may converge on or counterbalance nociceptor and spinal excitability, but their relationship to PGE2 should not be interpreted as a single validated linear pathway [55,56,57,58,59,60]. Arthroplasty may reduce the peripheral driver but also creates acute surgical nociception. Recovery therefore depends on both removal of the target-joint generator and the behaviour of the wider nociceptive system. Arrows represent plausible reinforcing pathways rather than obligatory or proven causal sequences. Selected central neuroimmune pathways are supported by non-arthritis pain models and are shown as mechanistic analogues rather than arthritis-specific human evidence.

Two trajectories often coexist. In one, inflammation is controlled but irreversible damage, instability, deformity, or abnormal loading maintains peripheral nociception. In the other, pain becomes less anatomically confined and increasingly influenced by distributed processing. Their relative contribution changes over time. A patient may move from inflammation-dominant pain to structural-concordant pain, then develop widespread hypersensitivity after years of nociceptive input.

Arthroplasty can remove diseased surfaces, correct alignment, restore stability, and reduce peripheral input. Replacement does not treat contralateral joint disease, lumbar stenosis, enthesitis, fibromyalgia symptoms, sleep apnoea, or depression. Surgery also introduces acute tissue injury and can interact with pre-existing vulnerability to persistent postsurgical pain [71]. Marked target-joint relief may therefore coexist with persistent global pain.

This bidirectional model avoids two errors: dismissing a structurally damaged joint because pain is widespread, and promising global pain resolution because the implant is technically successful. Features of distributed pain amplification (including, in some patients, a nociplastic component) identify uncertainty about the magnitude of joint-specific benefit and the risk of residual symptoms; they do not determine surgical eligibility.

## 6. Translational and Clinical Implications of Molecular and Neuroimmune Pain Mechanisms

### 6.1. Clinical Expression of Pain Amplification in Inflammatory Arthritis

Prevalence estimates are not directly comparable because fibromyalgia criteria, CSI thresholds, pain maps, and QST protocols differ. Across rheumatoid arthritis, psoriatic arthritis, and axial spondyloarthritis, widespread pain and fibromyalgia traits are consistently associated with higher pain, fatigue, disability, tender-joint counts, and patient global scores [31,41]. Their relation to objective inflammatory activity is weaker and more heterogeneous.

A 2023 systematic review and meta-analysis of 63 studies involving 13,035 patients underscored this measurement dependence; pooled prevalence estimates for questionnaire-defined pain sensitivity or neuropathic-like pain ranged from 31% with Douleur Neuropathique en 4 Questions (DN4) to 42% with CSI, with substantial between-study heterogeneity and inconsistent evidence that these constructs can predict treatment outcome [72]. A separate systematic review of QST in 27 inflammatory-rheumatic-disease studies found reduced pressure-pain thresholds in 12 out of 14 controlled rheumatoid-arthritis or spondyloarthritis studies, whereas temporal summation and conditioned pain modulation findings were inconsistent because of population heterogeneity and limited standardisation [73]. These syntheses support group-level sensory abnormalities while reinforcing that questionnaire or QST findings do not identify single-mechanism-defined subgroups in an individual patient. A common limitation across these studies is the lack of a genuinely personalised, integrative approach. Most analyses evaluate individual questionnaires, sensory measures, biomarkers, or group-level associations rather than combining structural pathology, objective inflammatory activity, pain distribution, sensory phenotype, comorbidity, and contextual factors to identify the dominant pain mechanisms in an individual patient. Consequently, population-level associations cannot yet be translated reliably into patient-specific phenotyping, prognosis, or perioperative decision-making.

Prospective rheumatoid-arthritis data now extend this evidence. In a 2026 cohort of 158 adults with newly diagnosed rheumatoid arthritis, higher baseline painDETECT scores were associated with worse bodily pain over 12 months after adjustment for baseline outcome, sociodemographic factors, and comorbidities, while no association with C-reactive protein was observed. In an embedded functional magnetic resonance imaging sub-study of 27 participants, higher painDETECT scores correlated with a greater activation in the left insula, dorsal anterior cingulate cortex, and left amygdala during evoked wrist pain [74]. These findings strengthen disease-specific prognostic evidence for neuropathic-like pain features, but painDETECT remains a screening measure and does not establish a pain mechanism or arthroplasty eligibility.

Rheumatoid arthritis provides the strongest disease-specific mechanistic evidence. Lower local and remote pressure-pain thresholds, enhanced reactivity, and altered inhibitory responses have been described, although effect sizes and anatomical patterns vary [32,33,34,35]. In clinically active rheumatoid arthritis, inflammatory variables contributed more to pain than QST indices, arguing against sensory amplification as a substitute explanation for uncontrolled disease [36]. Exploratory PBMC data also suggest molecular correlates of CSI-defined symptom burden in rheumatoid arthritis, with comparatively limited pro-inflammatory cytokine gene expression; however, the cross-sectional design, peripheral sampling, and questionnaire-defined grouping preclude mechanistic or perioperative prognostic interpretation [53]. Consistent with this apparent dissociation between pain-related abnormalities and conventional inflammatory activity, the 2025 exploratory multi-disease QST study identified somatosensory abnormalities and allodynia that were not associated with disease activity or inflammatory markers; however, its small disease-specific strata preclude prevalence claims [37]. Together, these findings support the possibility that altered pain processing may coexist with low or discordant conventional inflammatory activity, without establishing a single non-inflammatory pain mechanism. Taken together, these studies support multi-domain assessment but do not yet provide an individualised method for determining the relative contribution of inflammatory, structural, neuropathic, and nociplastic or amplification-related mechanisms in a given patient.

Psoriatic arthritis evidence remains dominated by cross-sectional questionnaires. CSI-defined symptom burden tracks pain, disability, sleep, mood, and composite disease activity [38,39,40]. Shared symptom content with correlated outcomes creates common-method variance and circularity. Axial spondyloarthritis studies similarly show that CSI scores combine sensory and psychosocial domains and may rise without a corresponding impairment in conditioned pain modulation [44]. These data support multi-domain assessment. They do not identify a mechanism-defined subgroup suitable for surgical exclusion.

### 6.2. Preoperative Pain Phenotypes and Total Knee Arthroplasty: Indirect Translational Evidence

The most mature perioperative evidence comes from knee osteoarthritis and is indirect. Recent arthroplasty-focused syntheses likewise emphasise the prognostic relevance of nociplastic and central-sensitisation features before hip or knee replacement, while underscoring that the available evidence remains largely observational and dominated by osteoarthritis populations [75,76]. Systematic reviews associate preoperative widespread pain, neuropathic-like symptoms, fibromyalgia survey scores, CSI-defined symptom burden, catastrophising, depression, anxiety, poor sleep, opioid exposure, and selected QST abnormalities with postoperative pain [77,78,79]. Baseline pain itself is also an established predictor of persistent postoperative pain, while residual confounding by baseline symptom severity, comorbidity, access, and treatment selection remains a recurrent limitation of this literature [77,78].

Questionnaire-defined phenotypes show the clearest association with an unfavourable absolute postoperative pain state [25,79,80]. A 2025 meta-analysis included 15 studies and 2385 patients. A preoperative painDETECT score of at least 13 was associated with a higher risk of chronic postoperative pain, defined in the meta-analysis as a pain-intensity score of at least 3/10 on either a visual analogue scale or a numerical rating scale at least three months after surgery (pooled risk ratio, 2.75; 95% confidence interval, 1.78–4.26) [25]. Function, satisfaction, and psychological outcomes were inconsistent, as several associations weakened after adjustment [25]. In a 2026 multi-centre cohort of 192 patients, each one-point increase in CSI score was associated with greater odds of failing to achieve a 10-point improvement in the Knee Injury and Osteoarthritis Outcome Score (KOOS) pain subscale at three months (adjusted odds ratio, 1.115; 95% confidence interval, 1.060–1.173) [80]. This continuous association is clinically informative, but it does not validate a diagnostic or prognostic cut-off or establish long-term predictive performance [80].

Cohorts using fibromyalgia survey scores similarly demonstrate a graded association rather than a simple threshold effect [26,27]. In one study, each one-point increase in the score raised the odds of failing to achieve at least 50% pain improvement by 17.8%, even among patients below the fibromyalgia diagnostic threshold [26]. The score also predicted higher acute opioid consumption after lower-extremity arthroplasty [27]. Studies combining CSI and painDETECT groups report cumulatively worse patient-reported outcomes, but the exposure categories remain questionnaire constructs rather than confirmed mechanisms [81].

QST findings are less stable across the studies [24,82,83,84]. Remote pressure sensitivity, enhanced temporal summation, and cuff-algometry profiles have predicted persistent pain in individual cohorts [82,83,84]. Other studies have found weak or non-independent associations, and the 2025 individual-participant-data meta-analysis did not support consistent person-level predictions across lower-limb osteoarthritis treatments [24]. QST therefore remains valuable for mechanistic research, but current evidence does not support its use as a stand-alone instrument for determining surgical eligibility [24,82,83,84].

Function and satisfaction require separate analysis. Expectations, contralateral disease, disability, mental health, rehabilitation, and complications influence satisfaction [85,86,87]. Preoperative opioid use is associated with worse postoperative patient-reported outcomes, although confounding is difficult to eliminate [88]. Sleep disturbance is a general risk marker for chronic postsurgical pain, but intervention evidence in arthroplasty remains limited [89]. Catastrophising predicts pain in some cohorts [90], yet brief cognitive-behavioural education did not improve pain or function in high-catastrophising total knee arthroplasty (TKA) candidates [91]. Prognostic association must not be mistaken for proof that a short preoperative treatment will remove the risk.

### 6.3. Total Hip and Other Joint Arthroplasty: Limits of Mechanistic Extrapolation

Persistent pain is clinically relevant after both total hip and total knee arthroplasty, although reported estimates vary according to procedure, definition, and follow-up. The 2025 updated meta-analysis estimated that at least 14% of recipients reported pain at six to twelve months after hip replacement; certainty was limited by heterogeneity and loss to follow-up [4]. A 2026 review of 15 studies identified several pain, disability, psychosocial, behavioural, and comorbidity predictors, but adjustment was inconsistent, and no model was validated [92]. Mixed hip and knee cohorts have associated widespread pain and fibromyalgia traits with poorer absolute analgesic outcomes [26,93]. CSI-defined symptom burden may decline after arthroplasty, becoming compatible with partial reversibility [94].

A retrospective longitudinal study from a single large United States academic centre included 6631 patients undergoing knee (46.6%), hip (31.2%), or shoulder (22.2%) arthroplasty. High-impact chronic pain was present in 45.3% of patients preoperatively. Postoperatively, 15.2% of the entire cohort had persistent high-impact chronic pain and 4.8% had new-onset high-impact chronic pain, giving an overall postoperative prevalence of 20.0% [95]. These transitions illustrate that a substantial population-level reduction in pain burden may coexist with persistent pain in some patients and new-onset high-impact pain in a smaller subgroup. Evidence for shoulder, elbow, ankle, and small-joint arthroplasty is sparse, heterogeneous, and mostly retrospective. Findings from TKA should not be transferred without explicit qualification.

### 6.4. Direct Translational Evidence in Inflammatory-Arthritis Arthroplasty

Direct evidence linking preoperative pain-mechanism phenotyping to arthroplasty outcome in inflammatory arthritis is extremely limited. Rheumatoid arthritis cohorts show that patients can obtain large improvements after hip or knee replacement while retaining worse absolute function or pain than osteoarthritis comparators [1,2]. Other modern TKA cohorts report similar improvements and high satisfaction, with differences emerging after adjustment for baseline status or in selected functional domains [3,96,97]. A 2026 observational cohort of 61 patients with rheumatoid arthritis undergoing primary TKA reported improvement across all KOOS domains and 85.2% postoperative satisfaction, but this did not assess preoperative pain-mechanism phenotyping [98]. None prospectively classified a preoperative nociplastic component using accepted clinical criteria or a validated perioperative phenotype.

Perioperative rheumatoid arthritis flares are common, but flare occurrence does not explain all poor one-year outcomes; higher baseline disease activity appears more consistently related to later pain and function [99,100]. Short-term hip outcomes in psoriatic arthritis can approximate those in osteoarthritis, although cohorts are modest and pain mechanisms have not been assessed [101,102]. Arthroplasty data in spondyloarthritis and in joints other than the hip or knee are insufficient for phenotype-specific prognostication [103,104].

We identified no inflammatory-arthritis study that has validated CSI; painDETECT, a fibromyalgia survey score; or a QST battery as an exclusion instrument. We identified no study showing that crossing a questionnaire threshold identifies patients who benefit more from delaying arthroplasty than from timely replacement. Table 2 therefore reports evidence volume and directness rather than a pseudo-quantitative certainty grade. A major evidence gap is a prospective inflammatory-arthritis cohort that measures target-joint attribution and distributed amplification before surgery and follows joint-specific and global outcomes separately.

### 6.5. Mechanism-Informed Preoperative Pain Phenotyping

A practical assessment has two tiers. The core tier belongs in routine rheumatology and arthroplasty practice. Recent British Society for Rheumatology guidance similarly recommends that poorly controlled pain in inflammatory arthritis be assessed for both disease activity and other causes rather than assumed to reflect inflammatory activity alone [105]. The history should distinguish target-joint from whole-body pain. A body map shows whether “knee pain” includes lumbar, hip, contralateral, or neuropathic territories. The clinician should establish whether familiar pain is reproduced by loading or joint-specific tasks; ask about rest pain, inflammatory stiffness, swelling, instability, locking, and referred symptoms; and identify the activity surgery is expected to restore.

Examination should document effusion, warmth, focal tenderness, deformity, instability, range restriction, crepitus, enthesitis, gait, muscle strength, and the manoeuvre that reproduces the patient’s usual pain. Swollen-joint assessment and review of systemic disease control remain essential. Plain radiography establishes structural severity and mechanical plausibility. Power Doppler ultrasonography is useful when examination and inflammatory indices conflict. Magnetic resonance imaging may be considered when there is a defined diagnostic uncertainty, such as occult osteonecrosis, insufficiency fracture, or unexplained symptoms; it should not be used as a general pain biomarker.

Alternative generators should be actively assessed and, where clinically indicated, excluded: lumbar radiculopathy or stenosis, peripheral neuropathy, complex regional pain syndrome, tendinopathy, enthesitis, trochanteric or periarticular pain, referred hip or spinal pain, vascular claudication, infection, crystal disease, and pain from another damaged joint. In selected cases of persistent diagnostic uncertainty—particularly when hip and spinal sources compete—a targeted image-guided intra-articular local-anaesthetic injection may be considered as an adjunct to attribution. Evidence for diagnostic use in arthroplasty decision-making is joint-specific and largely derived from hip cohorts; the result should not be treated as a definitive test or used alone to determine arthroplasty eligibility. A transient response should be interpreted within the broader clinical pattern [106].

Direct inflammatory-arthritis evidence nevertheless supports a local-anaesthetic challenge as a mechanistic probe of peripheral joint contribution. In a 2024 study, intra-articular lidocaine produced a greater immediate reduction in pain than saline, while higher residual pain after lidocaine was associated with high painDETECT scores, fibromyalgia, and lower remote pressure-pain thresholds [107]. Because the study was not conducted in an arthroplasty population and did not test prediction of postoperative benefit, these findings do not validate an injection response as a surgical selection threshold.

The same consultation should separately record potentially modifiable factors and recovery reserve. Potentially modifiable factors may include residual inflammatory activity, untreated sleep disorder, mood symptoms, catastrophising, fear of movement, opioid exposure, deconditioning, obesity, and unrealistic expectations. Recovery reserve includes frailty, sarcopenia, baseline physical function, neurological or musculoskeletal comorbidity, social support, adherence, and the practical capacity to participate in rehabilitation. These domains overlap but are not interchangeable; a patient may have several treatment targets and a low recovery reserve at the same time. Contemporary guidance should govern perioperative antirheumatic medication rather than the pain phenotype [108].

Instruments can standardise domains without making the diagnosis. The CSI is a prompt for deeper assessment. painDETECT or modified painDETECT records neuropathic-like qualities; a high score warrants neurological examination and differential diagnosis. The Widespread Pain Index, Symptom Severity Scale, and fibromyalgia survey score quantify pain distribution and associated symptoms. The Pain Catastrophizing Scale, Insomnia Severity Index, Hospital Anxiety and Depression Scale, and Patient-Reported Outcomes Measurement Information System, as fatigue measures, cover potentially modifiable or prognostic domains [109,110,111,112]. Scores should be interpreted continuously and in context.

The research tier can include local and remote pressure-pain thresholds, temporal summation, conditioned pain modulation, cuff algometry, actigraphy, and repeated sensory trajectories. Each samples a limited process. Equipment, training, variability, medication, and absent clinical cut-offs restrict routine use. Research testing is justified when it addresses a defined question or prospective validation. Multi-domain clinical phenotyping with selective specialist testing is preferable to universal QST. At present, circulating or synovial cytokines, NGF, autoantibodies, or other molecular markers should not be used to assign phenotypes or determine arthroplasty eligibility because we identified no validated perioperative threshold for this purpose [51,52,53,54,61,62,63,64,65,67].

### 6.6. The Target-Joint Attribution and Pain-Amplification Framework: A Translational Synthesis

Target-joint attribution is a structured clinical estimate of the likelihood that the joint considered for replacement is a dominant and surgically remediable source of pain and functional limitation. It is not synonymous with radiographic severity, inflammatory activity, or pain intensity. Attribution is strongest when anatomical, clinical, functional, imaging, and pain-distribution findings converge.

The framework starts with the clinical decision and draws on four evidence streams: direct inflammatory-arthritis arthroplasty data, disease-specific phenotyping, indirect osteoarthritis perioperative data, and mechanistic evidence. Elements were retained when observable and capable of changing attribution, preparation, prognosis, or outcome interpretation. The framework includes an initial differential-diagnostic checkpoint for neuropathic and extra-articular pain generators, followed, when target-joint attribution remains plausible, by four clinical phenotypes. These phenotypes are an expert synthesis and are not clusters derived from data; no weights or cut-offs have been assigned, and the model is unvalidated (Figure 2).

The purpose of phenotypes A–D is not to assign a fixed or mutually exclusive diagnosis but to provide a decision-oriented snapshot of the dominant clinical pattern at a given stage of assessment. Their value lies in identifying which issue should guide the next perioperative step: a structural-concordant pattern supports routine surgical planning; an inflammation-dominant pattern prioritises inflammatory optimisation and reassessment; a mixed joint-amplification pattern supports surgery when target-joint attribution remains strong but modifies counselling, preparation, and postoperative care; and a distributed pain-dominant pattern prompts closer reassessment of attribution and more explicit discussion of uncertainty in pain relief. When features overlap, the clinician should document the dominant current pattern together with the relevant coexisting modifiers rather than force an exclusive classification.

Domain 1 asks whether the target joint behaves as the dominant generator. Evidence includes anatomically concordant pain; reproducible loading or movement pain; joint-specific functional loss; advanced damage; deformity or instability; restricted range; local tenderness or effusion; synovitis; altered gait; and inability to perform tasks that specifically depend on that joint. A proportionate response to a local intervention may support attribution but is optional and never decisive alone.

Domain 2 characterises distributed pain amplification beyond the target joint. Relevant findings include widespread or multisite pain, regional spread, allodynia or hyperalgesia, remote pressure sensitivity, a marked mismatch between local pathology and global symptom burden, enhanced temporal summation, impaired conditioned pain modulation, unrefreshing sleep, fatigue, cognitive symptoms, variability, fibromyalgia traits, and co-occurring pain or symptom disorders. Because these findings are heterogeneous and many are self-reported or non-specific, convergence matters more than any single feature or score. The term distributed pain amplification is descriptive and does not imply a single neurophysiological mechanism.

Domain 3 describes preparation and recovery context and is explicitly multidimensional rather than a single continuum. It comprises two related but distinct components. The first is modifiability: factors for which a defined intervention could improve attribution, perioperative readiness, or participation in rehabilitation. The second is recovery reserve: the patient’s physiological, functional, psychological, and social capacity to tolerate surgery and participate in recovery. Because inflammatory arthritis is a systemic disease, recovery reserve should be assessed at the whole-patient level rather than inferred from the target joint or pain phenotype alone. Systemic disease burden may affect fatigue, physical capacity, multimorbidity, and overall rehabilitation tolerance, while immunomodulatory treatment introduces additional perioperative considerations related to disease control, infection risk, and flare prevention [47,108]. These systemic factors may influence perioperative readiness and postoperative recovery independently of whether the dominant pain pattern is structural, inflammatory, or amplification-related. Residual inflammatory activity, sleep disorder, depression, anxiety, catastrophising, fear of movement, opioid exposure, obesity, and deconditioning may be treatment targets in selected patients. Frailty, sarcopenia, multimorbidity, baseline physical function, social support, adherence, and rehabilitation capacity primarily inform reserve and perioperative planning, although some factors contribute to both components. A prognostic association does not establish modifiability, and low recovery reserve may coexist with many or few modifiable factors. Accordingly, phenotype assignment should complement, rather than replace, conventional whole-patient assessment of systemic inflammatory disease and perioperative medical risk.

Before phenotype assignment, the clinician should determine whether a neuropathic or extra-articular generator better explains the index symptoms. Radiculopathy, peripheral neuropathy, complex regional pain syndrome, tendinopathy, enthesitis, vascular disease, and referred spinal or hip pain form a differential-diagnostic checkpoint rather than a pain phenotype. When such a generator is suspected, it should be clarified and treated before assignment to phenotypes A–D, while recognising that an alternative generator may coexist with genuine target-joint pathology. Four recognisable phenotypes then follow.

Phenotype A, structural-concordant, combines advanced target-joint pathology, anatomically concordant pain, reproducible joint-specific limitation, and little widespread pain. The expected target-joint benefit is generally favourable when target-joint attribution is strong, subject to ordinary surgical risk.

Phenotype B, inflammation-dominant, has active local or systemic inflammatory disease (including synovitis, enthesitis, dactylitis, or axial inflammatory activity where relevant) and an uncertain contribution from irreversible damage. Inflammatory optimisation and planned reassessment take priority unless deformity, instability, or progressive deterioration makes delay harmful.

Phenotype C, the mixed target-joint and distributed pain-amplification phenotype, combines a secure structural indication and clear joint-specific symptoms with convergent features of pain amplification beyond the target joint. These may include widespread or multisite pain, regional pain spread, hypersensitivity, unrefreshing sleep, fatigue, cognitive symptoms, or fibromyalgia traits. These features do not by themselves establish nociplastic pain. Some patients within this phenotype may also meet accepted clinical criteria for nociplastic pain, but this is not required for classification. Arthroplasty is often reasonable when target-joint attribution remains high. Counselling should distinguish the expected relief of the joint-specific component from the greater probability of residual widespread or non-target pain and the continued need for non-joint treatment.

Phenotype D, distributed pain-dominant, has widespread symptoms, weak anatomical concordance, and little relation between the index pain and target-joint loading or function. The risk of a mismatch between surgery and the dominant symptom burden is greater. This phenotype is not an automatic reason to deny surgery. Progressive deformity, instability, irreversible loss of function, or impending structural failure may still justify replacement, with explicit acknowledgement that pain relief is less predictable. Optimisation should be time limited and followed by reassessment.

Phenotypes A–D overlap and may change over time, and an alternative pain generator may coexist with any of them. This dynamism is intentional rather than a limitation of the framework: phenotype assignment should be regarded as a time-specific clinical state and reassessed after treatment of modifiable factors or when the balance between inflammatory, structural, and distributed pain mechanisms changes. An inflammation-dominant patient may become structural-concordant after medical control; a mixed joint-amplification phenotype may become more favourable after interventions addressing sleep, opioid exposure, and rehabilitation capacity. Movement between phenotypes may therefore help structure reassessment and readiness for surgery, but should not be interpreted as a validated treatment-response metric. Table 3 translates the framework into a practical pathway.

A calibration example illustrates its use. A destroyed knee with reproducible weight-bearing pain, varus instability, and widespread tenderness may still carry high target-joint attribution; amplification changes counselling and support, not the indication. Similar radiographic damage with dermatomal leg pain, little knee-provoked pain, and neurological signs carries low current attribution and warrants investigation of the alternative generator first.

### 6.7. From Mechanism-Informed Attribution and Phenotype to Decision

Figure 3 organises eight sequential questions: Is there a valid structural and functional indication? Is inflammatory disease sufficiently controlled for safe surgery and reliable attribution? Are pain distribution and behaviour concordant with the target joint? Is a neuropathic or extra-articular generator likely to better explain the index symptoms? Is distributed amplification clinically important? Which of phenotypes A–D best describes the presentation? Which factors are potentially modifiable before surgery? What is the patient’s recovery reserve and capacity to participate in rehabilitation? The alternative-generator assessment is a pre-classification checkpoint rather than a fifth phenotype, and modifiability and recovery reserve are assessed separately.

Four decisions follow. Proceed when target-joint attribution is high and no major reversible impediment is present. Proceed with phenotype-informed optimisation when the indication is secure but modifiable factors or distributed pain increase residual risk. Defer briefly and reassess when uncontrolled inflammation or a defined treatment trial could materially change attribution or perioperative readiness. Investigate or treat an alternative generator when history, examination, or imaging points away from the target joint.

Timing must remain proportional to structural urgency and disability. Although developed for osteoarthritis and osteonecrosis rather than inflammatory arthritis, contemporary American College of Rheumatology/American Association of Hip and Knee Surgeons (ACR/AAHKS) guidance supports avoiding mandatory additional low-yield nonoperative treatment once an operative indication is established [113]. Extrapolation of this principle to inflammatory arthritis should be cautious and account for disease control, structural urgency, and disability. Phenotyping must not become a route to indefinite delay. Optimisation should have a mechanism-matched intervention, endpoint, and scheduled reassessment.

Shared decision-making requires two forecasts: expected change in target-joint pain and function, and expected residual global symptom burden. Surgery may be chosen for stability, mobility, deformity correction, or independence even when complete pain relief is unlikely. Severe radiographic damage does not compel replacement when symptoms arise mainly elsewhere.

### 6.8. Mechanism-Informed Preoperative Optimisation: Evidence, Uncertainty, and Proportionality

Preoperative optimisation should be mechanism-matched, time-limited, and directed at factors that plausibly affect target-joint attribution, perioperative readiness, or recovery rather than at questionnaire thresholds. Inflammatory activity and antirheumatic medication require coordinated rheumatology-surgical management [105,108]; treatment escalation should not be driven solely by tenderness or patient global scores when swelling and imaging do not support active synovitis [30,31,105]. Exercise and progressive strengthening can support function and general health [114], while defined sleep disorders, including suspected obstructive sleep apnoea, should be assessed according to established guidance [115]. Mood symptoms, catastrophising, fear of movement, opioid exposure, deconditioning, and metabolic factors may warrant targeted management, but their prognostic association does not establish that preoperative modification will improve arthroplasty outcomes [88,91].

Neuropathic symptoms require appropriate neurological evaluation, and pharmacological treatment should follow a recognised clinical indication rather than CSI or painDETECT thresholds. Duloxetine may be appropriate for a separate evidence-based indication, but evidence that it modifies arthroplasty outcomes in inflammatory arthritis remains insufficient [116]. The goal of optimisation is therefore to address clearly remediable barriers while avoiding indefinite delay when target-joint attribution and the structural indication are secure.

### 6.9. Measuring Mechanistically Relevant Outcomes After Arthroplasty

Outcome assessment should separate target-joint pain from widespread pain. Joint-specific pain and function should be accompanied by a pain map, global function, quality of life, satisfaction, expectations, analgesic use, disease activity, flare, complications, and participation.

Absolute postoperative status and change from baseline answer different questions. Minimal clinically important improvement identifies meaningful change, whereas the patient acceptable symptom state asks whether the final state is satisfactory [117,118]. Longitudinal trajectories reveal early recovery, delayed improvement, and persistent pain more accurately than a single endpoint [119,120]. Chronic postsurgical pain should be defined according to International Classification of Diseases, 11th Revision (ICD-11) criteria: pain that develops or increases after surgery, persists for at least 3 months, is localised to the surgical field or an anatomically plausible referral territory, and is not better explained by a surgical complication or pre-existing pain condition [15]. It should not be inferred from any pain reported after surgery.

An apparently poor result has at least four meanings: no improvement in target-joint pain; meaningful target-joint relief with persistent widespread pain; clinically important improvement accompanied by unmet expectations; or pain caused by infection, loosening, instability, fracture, malalignment, nerve injury, or another complication. Conflating these states misclassifies the surgical effect and obscures the treatment still required.

## 7. Molecular and Translational Research Agenda

A primary research priority is a prospective molecular-validation programme in inflammatory-arthritis arthroplasty cohorts. Candidate measures could include synovial-fluid and circulating inflammatory mediators; neurotrophin signalling, such as NGF; GDNF family ligands, such as ARTN/NRTN; and TAFA4-associated signalling, as well as exploratory markers of neuroimmune activation, sampled alongside pain maps and sensory phenotyping [51,52,53,54,55,56,57,58,59,60,61,62,63,64,65,66,67]. For clinical translation, candidate neuroimmune markers should first be demonstrably measurable and reproducible in peripheral blood or another clinically accessible biofluid, and the relationship between that peripheral or fluid-phase signal and pathway activity in the relevant joint or neural compartment should be established. This is especially important because circulating concentrations may not reflect the joint or neural microenvironment; paired serum and cerebrospinal-fluid data in chronic pain illustrate that central neuroimmune activation can occur without corresponding systemic inflammation [66]. Biomarker studies should then test incremental predictive value rather than simple cross-sectional correlation. Paired preoperative and postoperative sampling could determine whether removal of a dominant peripheral generator is accompanied by changes in molecular signals and whether those changes track target-joint pain, remote hypersensitivity, or both. Multi-omic approaches may eventually be useful, but any signature should undergo assay standardisation, internal validation, external validation, calibration, and decision-analytic testing before clinical use [121,122,123].

Clinical validation should proceed in parallel through prospective inflammatory-arthritis arthroplasty cohorts that assess target-joint attribution and distributed pain features before surgery, rather than further reliance on isolated cross-sectional estimates of CSI-defined symptom burden. Consecutive rheumatoid arthritis, psoriatic arthritis, and spondyloarthritis cohorts should complete a minimum preoperative dataset covering structural indication, objective inflammation, target-joint concordance, pain map, fibromyalgia traits, neuropathic-like symptoms, sleep, fatigue, mood, opioid exposure, frailty, expectations, recovery reserve, and rehabilitation capacity. A nested subgroup could undergo QST and actigraphy.

This clinical-validation priority aligns closely with the first research question in the British Society for Rheumatology’s 2026 guidance, which calls for disease-specific tools able to distinguish pain drivers such as synovitis, sensitisation, and mechanical factors [105]. The present framework should therefore be tested as a candidate structure for such attribution rather than treated as an already validated solution.

Follow-up at approximately 6 weeks, 3 months, 6 months, 12 months, and 24 months should measure target-joint pain, widespread pain, joint-specific and global function, acceptable symptom state, chronic postsurgical pain, satisfaction, opioid use, disease flare, complications, and participation. The mechanistic question is whether removal of the peripheral generator changes local and remote sensory processing and whether that change mediates clinical recovery. Table 4 summarises the translational status of the principal candidate pathways discussed in this review.

The proposed phenotypes A–D should be derived transparently and validated externally across sex, age, ethnicity, socioeconomic position, healthcare system, and joint type. Neuropathic and extra-articular pain generators should be recorded as separate, potentially coexisting diagnoses rather than forced into the phenotype structure. Prediction-model studies should address calibration, missing data, external validation, and clinical utility; current reporting and risk-of-bias guidance includes Transparent Reporting of a Multivariable Prediction Model for Individual Prognosis or Diagnosis + Artificial Intelligence (TRIPOD + AI) and Prediction Model Risk of Bias Assessment Tool + Artificial Intelligence (PROBAST + AI), while decision-curve analysis can assess net benefit [121,122,123]. Phenotype-guided trials should compare time-limited, mechanism-matched preparation with standard care. Patient-partnered studies should test whether explicit attribution and residual-risk counselling improve understanding and decisions.

Emerging data suggest that future phenotyping may also need to move beyond conventional pain-evoked QST. Waller et al. assessed experimental visual sensitivity across hip osteoarthritis, rheumatoid arthritis, psoriatic arthritis, chronic pelvic pain, fibromyalgia, and pain-free controls. In a pooled analysis of the osteoarthritis and chronic-pelvic-pain surgical cohorts, higher fibromyalgia survey severity and greater visual sensitivity predicted non-response six months after surgery, whereas pressure-pain sensitivity did not [124]. This is not direct validation in inflammatory-arthritis arthroplasty, but it supports testing non-painful sensory markers alongside established clinical and QST domains instead of assuming that pressure sensitivity alone captures distributed amplification.

Perioperative outcome phenotyping should also distinguish pain response from procedure-related safety. In a 2026 national database study of 593,013 total hip arthroplasties, including 1445 patients with psoriatic arthritis, psoriatic arthritis was associated with higher odds of 90-day superficial and deep surgical-site infection and sepsis, as well as a modestly higher two-year all-cause revision risk [125]. These data do not inform pain-mechanism classification, but they reinforce that favourable pain/function trajectories and pain-phenotype assessment should not be conflated with the separate perioperative complication profile of inflammatory arthritis.

## 8. Limitations

This review has several limitations. Although the literature search was structured and conducted across multiple databases, it was designed to support a critical narrative synthesis and should not be considered exhaustive. Study selection involved author judgement, and relevant publications may have been missed, particularly because only English-language literature was considered. A formal study-level risk-of-bias assessment and certainty grading were not performed. The available evidence was heterogeneous in populations, pain definitions, measurement instruments, outcome thresholds, follow-up periods, and covariate adjustment. Direct evidence linking preoperative pain-mechanism phenotyping with arthroplasty outcomes in inflammatory arthritis was particularly sparse. The molecular discussion also draws on preclinical arthritis models and, for selected central neuroimmune pathways, non-arthritis pain models; these data support biological plausibility but cannot establish human perioperative biomarker validity or treatment effects. Much of the perioperative evidence was therefore extrapolated from osteoarthritis, predominantly total knee arthroplasty, which limits direct applicability to inflammatory arthritis and to other joints. The proposed framework represents an expert conceptual synthesis and has not been empirically derived, prospectively validated, or shown to improve clinical outcomes. Its components and clinical utility require evaluation in prospective inflammatory-arthritis arthroplasty cohorts.

## 9. Conclusions

Persistent pain in inflammatory arthritis can arise from interacting cytokine, prostaglandin, neurotrophin, immune-complex, type 1 interferon, and spinal neuron–glia pathways that alter nociceptor gain and central processing. These mechanisms provide biological plausibility for discordance between inflammatory activity, structural joint damage, and symptom distribution, but no molecular marker is currently validated for perioperative arthroplasty stratification in inflammatory arthritis [51,52,54,61,62,63,64,65,67]. Clinically, arthroplasty selection therefore still requires structural damage, inflammatory activity, alternative pain generators, and altered nociceptive processing to be considered together. No questionnaire can determine whether a joint is surgically remediable, and features of distributed pain amplification, with or without clinically classified nociplastic pain, neither invalidate a genuine peripheral source nor guarantee a poor result; they modify prognosis, preparation, and interpretation of outcomes.

The Target-Joint Attribution and Pain-Amplification Framework makes three domains explicit: target-joint attribution, the burden of distributed pain-amplification features, and a multidimensional preparation-and-recovery domain that separates potentially modifiable factors from recovery reserve. Its purpose is to integrate structural, clinical, molecular-mechanistic, and contextual reasoning to forecast what surgery can address, what may remain, and what should be treated alongside the operation. A structurally indicated replacement may remain appropriate in the presence of widespread pain, provided residual risk is discussed. Weak anatomical concordance should prompt a time-limited search for alternative generators before irreversible treatment. The framework proposed is unvalidated; it is a clinical reasoning aid and not a formal decision rule.

## Figures and Tables

**Figure 1:**
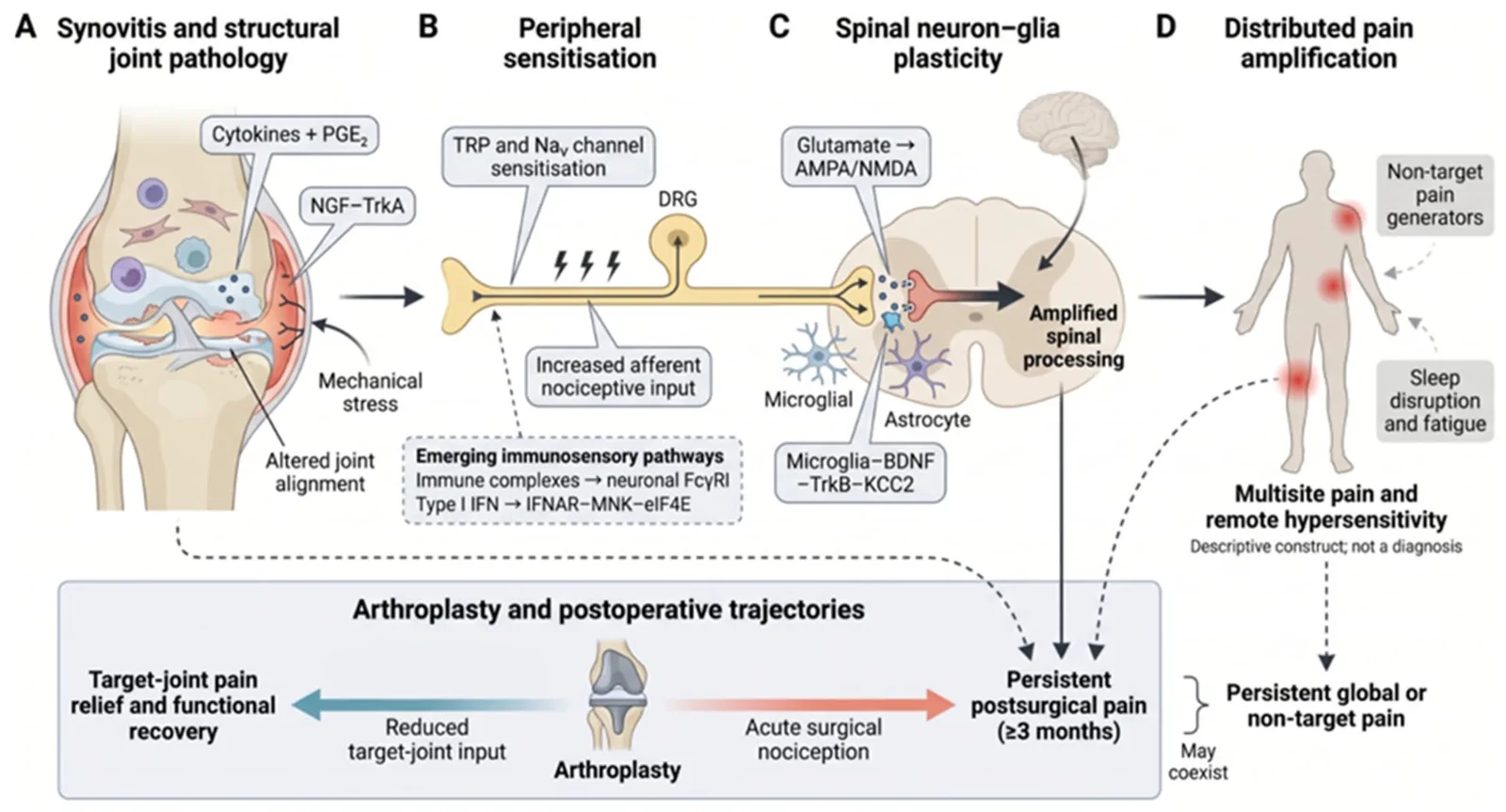
**From synovitis to persistent postsurgical pain: peripheral and neuroimmune pathways.** The figure shows representative rather than exhaustive pathways; additional candidate modulators discussed in the text include ARTN/GFRα3–RET and NRTN/GFRα2–RET signalling at peripheral nociceptors and TAFA4-associated signalling as a potentially counter-regulatory pathway. These mechanisms are predominantly supported by preclinical evidence and are not validated perioperative biomarkers. Abbreviations: AMPA, α-amino-3-hydroxy-5-methyl-4-isoxazolepropionic acid receptor; BDNF, brain-derived neurotrophic factor; DRG, dorsal-root ganglion; eIF4E, eukaryotic translation initiation factor 4E; FcγRI, Fc gamma receptor I; IFN, interferon; IFNAR, interferon alpha/beta receptor; KCC2, potassium-chloride cotransporter 2; MNK (MNK1/MNK2), MAP kinase-interacting serine/threonine-protein kinases 1 and 2; Nav, voltage-gated sodium channels; NGF, nerve growth factor; NMDA, N-methyl-D-aspartate receptor; PGE2, prostaglandin E2; TRP, transient receptor potential channels; TrkA, tropomyosin receptor kinase A; TrkB, tropomyosin receptor kinase B; ARTN, artemin; FPR1, formyl peptide receptor 1; GDNF, glial cell line-derived neurotrophic factor; GFRα2/3, GDNF family receptor alpha 2/3; LRP, low-density lipoprotein receptor-related protein; NRTN, neurturin; PSPN, persephin; RET, rearranged during transfection receptor tyrosine kinase; TAFA4, TAFA chemokine-like family member 4.

**Figure 2:**
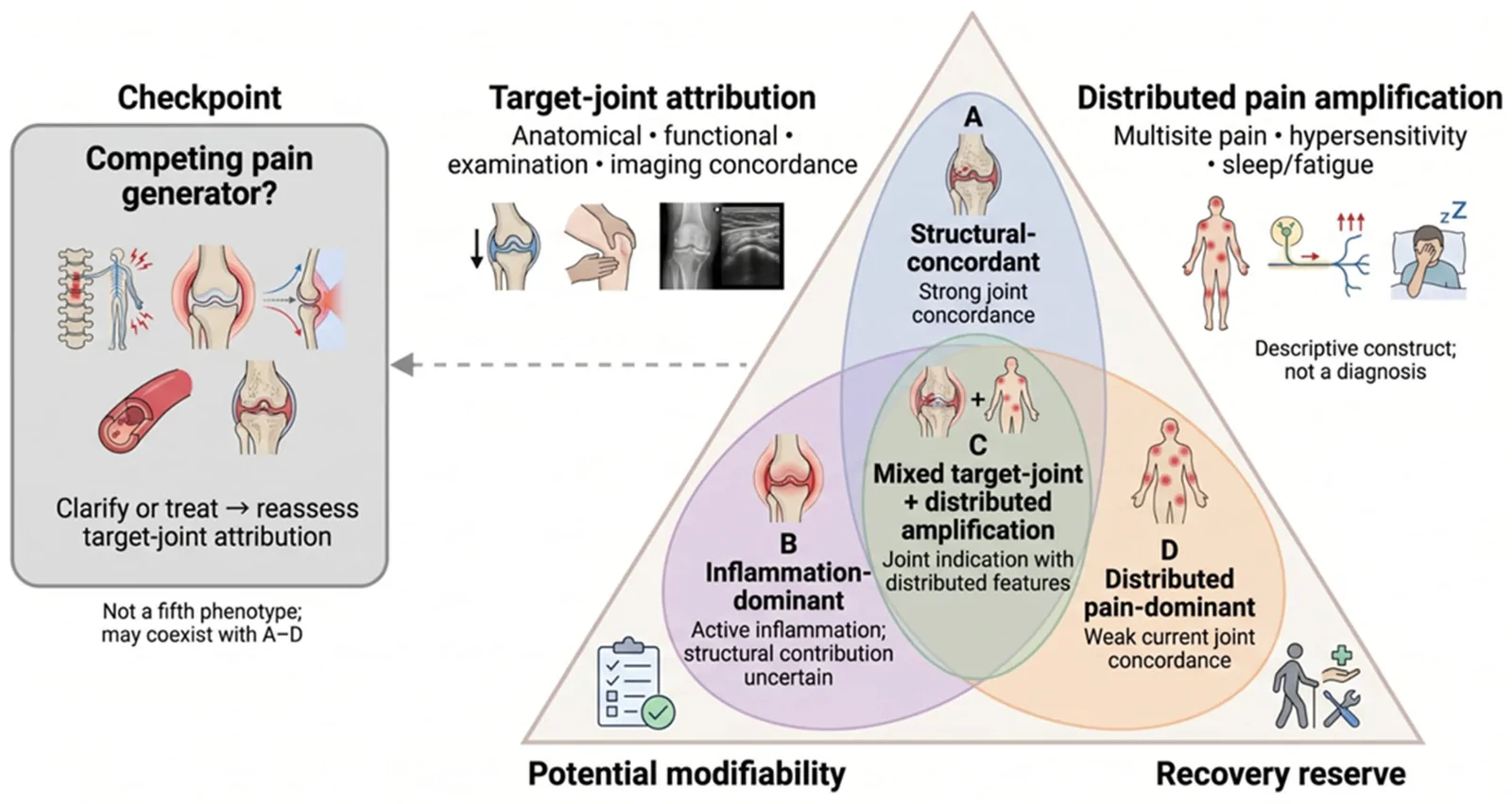
**The three-domain arthroplasty assessment framework.** Target-joint attribution is estimated from anatomical, functional, examination, and imaging concordance after assessment of competing pain generators. Distributed pain amplification modifies the residual-pain forecast, whereas preparation and recovery context informs readiness and rehabilitation planning. The diagram is schematic: spatial positions are non-quantitative, phenotypes may overlap, and the triangle does not represent validated axes or cut-offs. The phenotypes represent dynamic clinical states intended to guide assessment, preparation, counselling, and reassessment rather than mutually exclusive diagnostic categories. The framework represents the clinical translation of the cellular and molecular mechanisms discussed in Section 5 and Figure 1; it is not intended as a cellular or molecular pathway map.

**Figure 3:**
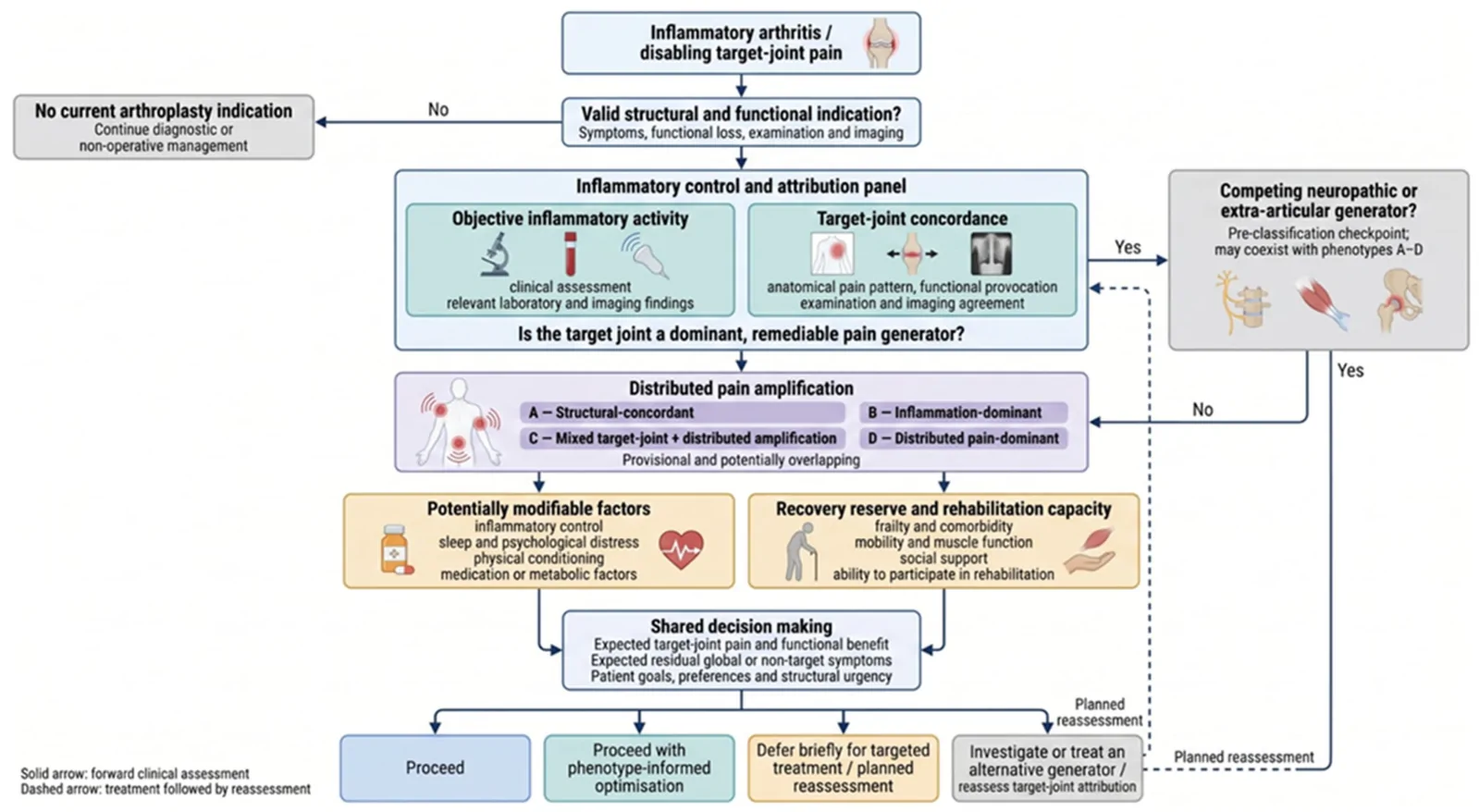
**Mechanism-informed preoperative decision pathway.** A valid structural indication is followed by assessment of inflammatory control and target-joint concordance. Suspected neuropathic or extra-articular generators trigger a pre-classification diagnostic checkpoint. Distributed amplification is then assessed, followed by provisional assignment to phenotypes A–D, identification of potentially modifiable factors, and assessment of recovery reserve and rehabilitation capacity. The resulting action is proportionate: proceed; proceed with phenotype-informed optimisation; defer briefly for targeted treatment and planned reassessment; or investigate or treat an alternative generator. After optimisation or treatment of an alternative generator, the pathway returns to reassessment of target-joint attribution rather than ending the decision process.

**Table 1:** Pain terminology and assessment tools relevant to arthroplasty in inflammatory arthritis.

Construct	Clinical Meaning	Representative Assessment Methods	What the Method Measures	What It Does Not Establish	Principal Perioperative Evidence	Major Limitations
Nociceptive inflammatory pain	Pain related to active synovial or entheseal inflammation and inflammatory mediator activity.	History, swollen-joint examination, inflammatory markers, power Doppler ultrasonography; magnetic resonance imaging selectively.	Evidence of inflammatory activity and local concordance.	That all pain is inflammatory; absent synovial signal does not exclude a structural, extra-synovial, or referred pain generator.	Direct relevance to inflammatory arthritis; perioperative prognostic evidence is limited.	Activity varies over time; treatment and glucocorticoids may suppress findings.
Structural nociceptive pain	Pain generated by damaged, deformed, unstable, or abnormally loaded joint tissues.	Loading pattern, joint-specific tasks, examination, radiography, and alignment and stability assessments.	Anatomical and functional plausibility of a remediable joint source.	That radiographic severity equals pain severity or predicts complete relief.	Structural and functional indications remain the foundation of arthroplasty.	Multisite disease and referred pain can reduce attribution.
Peripheral sensitisation	Increased responsiveness of peripheral nociceptors near injured or inflamed tissues.	Local tenderness, primary hyperalgesia, local pressure-pain threshold.	Local sensory gain close to the joint.	Central sensitisation or nociplastic pain.	Mechanistically plausible; routine prognostic validation is limited.	Influenced by inflammation, testing site, medication, and examiner technique.
Neuropathic pain	Pain caused by a lesion or disease of the somatosensory system.	Neuroanatomical history, sensory examination, nerve studies, or imaging when indicated.	Graded evidence of a somatosensory lesion or disease [12].	That the target joint is not painful or arthroplasty is contraindicated.	Radiculopathy and neuropathy may explain residual symptoms or mimic joint pain.	Mixed states are common; confirmatory tests may be normal or unavailable.
Neuropathic-like symptoms	Burning, electric, tingling, numb, or evoked qualities without a confirmed lesion.	PainDETECT or modified painDETECT plus neurological assessment.	A symptom pattern that warrants differential diagnosis.	Neuropathic pain or nociplastic pain [21].	Associated with chronic pain after total knee arthroplasty in indirect osteoarthritis evidence [25].	Self-report overlaps with severe nociceptive pain; thresholds are not exclusion rules.
Central sensitisation	Increased responsiveness of central nociceptive neurons to normal or subthreshold input.	Quantitative sensory testing patterns, experimental paradigms, and convergent clinical inference.	Selected features of sensory processing.	A diagnosis from the Central Sensitization Inventory or one sensory test [16,18,20].	Selected abnormalities are prognostic in some knee cohorts; individual prediction is inconsistent [24].	No gold standard; protocol and clinical context materially affect results.
Nociplastic pain	Pain arising from altered nociception when nociceptive or neuropathic mechanisms do not fully explain the presentation.	Clinical criteria, pain distribution, hypersensitivity, symptom context, and exclusion of better explanations.	A mechanism-informed clinical phenotype [11].	Fibromyalgia, psychological distress, or absence of a concurrent peripheral generator.	Indirect evidence links features commonly associated with nociplastic pain or distributed pain amplification to residual pain; direct inflammatory-arthritis data are sparse.	Mixed mechanisms and uncertain individual attribution.
Fibromyalgia traits	A continuum of widespread pain with fatigue, sleep, and cognitive symptoms.	Widespread Pain Index, Symptom Severity Scale, and fibromyalgia survey score.	Pain distribution and associated symptom burden [13].	A single pain mechanism or absence of a peripheral generator.	Predicts worse absolute analgesic outcomes and opioid use in osteoarthritis arthroplasty cohorts [26,27].	Threshold effects and overlap with inflammatory symptoms.
Chronic postsurgical pain	Pain developing or increasing after surgery and persisting beyond the expected healing process, defined as at least 3 months after surgery.	Standardised postoperative history, site and distribution mapping, and complication work-up.	Persistence and relationship to surgery [15].	Failure of implant surgery without exclusion of infection, mechanical failure, or another cause.	Established outcome after hip and knee replacement [4].	Definitions, time points, and anatomical attribution vary.

Note. Questionnaire scores and sensory-test findings support, but do not replace, clinical attribution.

**Table 2:** Evidence map for pain-amplification phenotypes and arthroplasty outcomes.

Population	Evidence for Pain Amplification	Association with Inflammatory Activity	Evidence for Postoperative Pain	Evidence for Function	Evidence for Satisfaction	Directness for Inflammatory Arthritis/Principal Limitations
Rheumatoid arthritis	Moderate disease-specific evidence for widespread pain, fibromyalgia traits, and sensory hypersensitivity [31,32].	Variable; active inflammation may dominate, while tenderness and patient global scores can reflect amplification [36].	Limited direct outcome evidence; preoperative mechanism phenotyping is rarely measured [1,2,3,98].	Meaningful improvement is common, with residual absolute impairment in some cohorts [96,97].	Very limited phenotype-specific evidence.	Direct for disease and surgery, not for mechanism-based prediction; confounding by baseline severity and historical treatment era.
Psoriatic arthritis	Limited-to-moderate cross-sectional evidence, mainly questionnaire-defined [38,39,40].	Patient-reported activity correlates strongly; independence from objective inflammation remains uncertain.	Very limited; short-term hip outcomes may resemble osteoarthritis [101].	Limited.	Very limited.	Direct disease evidence but minimal perioperative phenotyping; peripheral, axial, entheseal, and metabolic heterogeneity.
Axial spondyloarthritis	Moderate disease-specific evidence for widespread pain, fibromyalgia traits, and sensory differences [37,41,44].	Patient-reported activity is affected; questionnaire scores do not map cleanly onto inflammation.	Very limited for arthroplasty.	Very limited.	No robust phenotype-specific evidence.	Disease-specific phenotyping exists; perioperative directness is very low and hip-specific data predominate.
Osteoarthritis undergoing total knee arthroplasty	High volume of questionnaire and sensory testing studies.	Not applicable to systemic inflammatory activity; local synovitis may contribute.	Moderate evidence for worse absolute pain, with neuropathic-like or distributed phenotypes [25,77,79,80].	Moderate but heterogeneous; quantitative sensory testing prediction is inconsistent [24].	Moderate association in selected cohorts.	Indirect only for inflammatory arthritis; heterogeneous definitions, residual confounding, and no validated exclusion threshold.
Osteoarthritis undergoing total hip arthroplasty	Moderate evidence, often pooled with knee cohorts.	Not applicable to systemic inflammatory activity.	Limited-to-moderate evidence for residual pain and candidate biopsychosocial predictors [4,92].	Limited-to-moderate evidence.	Limited evidence.	Indirect only; fewer hip-specific studies and frequent pooling with knee replacement.
Other arthroplasty populations	Limited and joint-specific.	Depends on the underlying disease.	Very limited.	Very limited.	Very limited.	Indirect and sparse; biomechanics, failure modes, and outcome instruments differ by joint [104].

Note. Ratings are narrative judgements based on volume, directness, consistency, and adjustment quality; they are not formal Grading of Recommendations Assessment, Development and Evaluation (GRADE) certainty ratings. “High” denotes a large and reasonably consistent evidence base, “moderate” a recurrent but heterogeneous association, “limited” sparse or inconsistent data, and “very limited” isolated or non-phenotyped evidence. Abbreviations: GRADE, Grading of Recommendations Assessment, Development and Evaluation. The clinical phenotype categories in this evidence map should not be interpreted as molecularly defined subgroups. Candidate pathways including PGE2, GDNF family ligand signalling, and TAFA4-associated modulation may contribute to pain amplification, but no pathway currently maps one-to-one onto a clinical phenotype or predicts arthroplasty outcomes.

**Table 3:** Attribution- and phenotype-informed arthroplasty pathway.

Clinical Category	Dominant Findings	Additional Assessment	Preoperative Management	Expected Surgical Benefit	Residual Risk	Counselling Priorities
Pre-classification checkpoint: alternative generator suspected	Radiculopathy, peripheral neuropathy, complex regional pain syndrome, tendinopathy, enthesitis, referred spinal or hip pain, or vascular pain that may better explain the index symptoms.	Focused neurological, spinal, vascular, or periarticular evaluation; investigations as indicated; reassessment of target-joint concordance.	Investigate and treat the alternative source, then reassess target-joint attribution before phenotype assignment.	Not estimable until the target-joint contribution is clarified.	Persistent symptoms despite technically successful replacement; delayed diagnosis of the dominant pain generator.	Explain diagnostic uncertainty, the possibility of coexisting pain generators, and the rationale for staged assessment and treatment.
A. Structural-concordant	Advanced target-joint damage; anatomical and loading concordance; joint-specific functional loss; little widespread pain.	Routine exclusion of alternative generators; recovery reserve.	Standard medical and surgical optimisation; rehabilitation plan.	Expected target-joint pain and functional benefit is generally favourable.	Usual surgical and rehabilitation risks; pain elsewhere may persist.	Describe realistic recovery and complications without promising complete pain abolition.
B. Inflammation-dominant	Active local or systemic inflammatory disease (including synovitis, enthesitis, dactylitis, or axial inflammatory activity where relevant); contribution of irreversible damage remains uncertain.	Objective inflammatory assessment; imaging selectively; medication review.	Optimise inflammation and perioperative treatment; planned reassessment.	Uncertain until the inflammatory component is clarified; structural indications may remain.	Flare, infection, ongoing inflammatory pain, and delay-related deterioration.	Set a defined reassessment date; avoid indefinite delay when deformity or functional loss progresses.
C. Mixed joint-amplification	Convincing structural indication and joint-concordant symptoms, with convergent features of distributed pain amplification, such as widespread or multisite pain, hypersensitivity, fatigue, or unrefreshing sleep. Nociplastic pain may be present but is not assumed.	Pain map; assessment of pain distribution and target-joint concordance; symptom instruments; sleep, mood, fatigue, and opioid exposure; sensory testing only when decision-relevant; formal nociplastic-pain criteria when clinically indicated.	Where target-joint attribution remains high, proceed with time-limited multimodal preparation and coordinated postoperative care.	Often substantial target-joint benefit.	Higher residual pain, slower recovery, unmet global expectations, and continued analgesic need.	Separate expected joint relief from global pain and plan continuing non-joint treatment.
D. Distributed pain-dominant	Widespread burden; weak anatomical concordance; symptoms poorly related to target-joint loading or function.	Reassess alternative generators and structural urgency; specialist pain assessment where useful.	Time-limited, mechanism-matched treatment, then reassess; surgery may remain justified for stability or function.	Pain benefit uncertain; mechanical or functional benefit may remain.	Greater risk that the dominant symptoms persist.	Do not promise global pain relief; do not use the phenotype as automatic denial.

Note. Alternative pain generators are assessed before phenotype assignment and may coexist with any of phenotypes A–D. Phenotypes A–D overlap and may change after inflammatory treatment, rehabilitation, or clarification of a competing generator. None of phenotypes A–D is an automatic exclusion category. Expected-benefit and residual-risk descriptors are qualitative expert-inference statements and should not be interpreted as validated probabilities.

**Table 4:** Translational status of candidate molecular and neuroimmune pathways relevant to pain phenotyping.

Pathway	Evidence Compartment	Key Evidence Relevant to Pain	Candidate Measurement	Current Perioperative Status
Cytokines and PGE2	Human inflammatory-arthritis evidence plus preclinical mechanistic studies	TNF-α, IL-1β, IL-6, IL-17, and prostaglandin signalling can sensitise nociceptors; pain intensity need not track a single systemic inflammatory marker [51,52].	Circulating or synovial cytokines and lipid-mediators in research protocols	Research only; no validated threshold or classifier for arthroplasty benefit.
GDNF family ligands (ARTN/NRTN; PSPN evidence limited)	Preclinical inflammatory bone and osteoarthritis pain studies plus mechanistic reviews	ARTN/GFRα3–RET and NRTN/GFRα2–RET signalling can activate or sensitise peripheral and bone afferent neurons; ARTN may converge with PGE2 at TRPV1-related nociceptor sensitisation, whereas direct pain evidence for PSPN is limited [55,56,57,58].	ARTN/NRTN ligand concentrations and GFRα/RET pathway measures in tissue, synovial fluid, or circulating samples in research protocols	Mechanistic candidate only; no validated perioperative threshold, phenotype assignment rule, or arthroplasty classifier.
NGF-TrkA and ion-channel sensitisation	Preclinical and translational bone/joint-pain literature	NGF-TrkA signalling can increase nociceptor gain and alter TRPV1-related transduction while interacting with tissue remodelling [54].	NGF/pathway assays combined with clinical or sensory phenotyping	No validated perioperative threshold; therapeutic translation cannot be assumed from mechanistic association.
Immune-complex/Fcγ-receptor signalling	Preclinical arthritis models	Cartilage-binding immune complexes and neuronal Fcγ-receptor signalling can produce pain partly dissociated from histological inflammation [61,62].	Autoantibody/immune-complex profiles; experimental receptor-pathway measures	Biological plausibility only for perioperative use; no validated clinical classifier.
Type 1 IFN-IFNAR-MNK1/MNK2-eIF4E	Autoantibody-induced arthritis plus human rheumatoid arthritis dorsal-root-ganglion translational data	Persistent type 1 IFN signalling can sustain sensory-neuron hyperexcitability across inflammatory and post-inflammatory phases [67].	Type 1 interferons and pathway-activation markers such as phosphorylated eukaryotic translation initiation factor 4E (p-eIF4E) in research samples	Research only; no arthroplasty validation or clinically deployable perioperative threshold.
TAFA4-associated signalling	Preclinical inflammatory, postoperative, and neuropathic pain models	TAFA4 can reduce mechanical hypersensitivity and modulate spinal neuronal excitability through LRP-dependent mechanisms; FPR1/neurexin-associated signalling provides an additional neuroimmune interface [59,60].	Exploratory TAFA4/FPR1/LRP pathway assays in tissue or accessible biofluids; no established clinical assay	Experimental and potentially counter-regulatory; no validated arthroplasty biomarker or phenotype-specific classifier.
Spinal neuron–glia plasticity	Arthritis models plus selected non-arthritis pain models	Glutamatergic, purinergic, p38 MAPK, BDNF-TrkB/KCC2, and related glial pathways provide biological plausibility for distributed amplification [63,64,65].	Cerebrospinal fluid or experimental neural/glial pathway measures; peripheral-blood surrogates would require demonstration of correspondence with central pathway activity.	Mechanistic evidence only; no validated, clinically accessible peripheral biomarker.
PBMC gene-expression and multi-omic signatures	Exploratory cross-sectional human RA data	CSI-defined symptom groups have shown peripheral gene-expression differences, but the sampling compartment and design preclude mechanistic attribution [53].	PBMC transcriptomics or broader multi-omic panels linked to longitudinal phenotyping	Exploratory only; requires assay standardisation, incremental-value testing, internal and external validation.

Abbreviations: BDNF, brain-derived neurotrophic factor; CSI, Central Sensitization Inventory; eIF4E, eukaryotic translation initiation factor 4E; Fcγ receptor, Fc gamma receptor; IFN, interferon; IFNAR, interferon alpha/beta receptor; IL-1β, interleukin-1 beta; IL-6, interleukin-6; IL-17, interleukin-17; KCC2, potassium-chloride cotransporter 2; MAPK, mitogen-activated protein kinase; MNK1/MNK2, MAP kinase-interacting serine/threonine-protein kinases 1 and 2; NGF, nerve growth factor; PBMC, peripheral blood mononuclear cell; PGE2, prostaglandin E2; p-eIF4E, phosphorylated eukaryotic translation initiation factor 4E; RA, rheumatoid arthritis; TNF-α, tumour necrosis factor alpha; TrkA, tropomyosin receptor kinase A; TrkB, tropomyosin receptor kinase B; TRPV1, transient receptor potential vanilloid 1.

## Data Availability

No new data were created or analysed in this study. Data sharing is not applicable to this article.

## Abbreviations

The following abbreviations are used in this manuscript:

ACR/AAHKS	American College of Rheumatology/American Association of Hip and Knee Surgeons
AMPA	α-amino-3-hydroxy-5-methyl-4-isoxazolepropionic acid receptor
ARTN	Artemin
ATP	Adenosine triphosphate
BDNF	Brain-derived neurotrophic factor
CSI	Central Sensitization Inventory
CX3CR1	C-X3-C motif chemokine receptor 1
DN4	Douleur Neuropathique en 4 Questions
eIF4E	Eukaryotic translation initiation factor 4E
FcγRI	Fc gamma receptor I
FPR1	Formyl peptide receptor 1
GDNF	Glial cell line-derived neurotrophic factor
GFRα2	GDNF family receptor alpha 2
GFRα3	GDNF family receptor alpha 3
GFRα4	GDNF family receptor alpha 4
GRADE	Grading of Recommendations Assessment, Development and Evaluation
HMGB1	High-mobility group box 1
IASP	International Association for the Study of Pain
ICD-11	International Classification of Diseases, 11th Revision
IFN	Interferon
IFN-α/β	Interferon alpha/beta
IFNAR	Interferon alpha/beta receptor
IL-17	Interleukin-17
IL-1β	Interleukin-1 beta
IL-6	Interleukin-6
KCC2	Potassium-chloride cotransporter 2
KOOS	Knee Injury and Osteoarthritis Outcome Score
LRP	Low-density lipoprotein receptor-related protein
MAPK	Mitogen-activated protein kinase
MNK1/MNK2	MAP kinase-interacting serine/threonine-protein kinases 1 and 2
MRI	Magnetic resonance imaging
NaV1.8/1.9	Voltage-gated sodium channel subtypes NaV1.8 and NaV1.9
NF-κB	Nuclear factor kappa B
NGF	Nerve growth factor
NMDA	N-methyl-D-aspartate receptor
NRTN	Neurturin
p-eIF4E	Phosphorylated eukaryotic translation initiation factor 4E
PBMC	Peripheral blood mononuclear cell
PGE2	Prostaglandin E2
PROBAST + AI	Prediction Model Risk of Bias Assessment Tool + Artificial Intelligence
PSPN	Persephin
QST	Quantitative sensory testing
RA	Rheumatoid arthritis
RET	Rearranged during transfection receptor tyrosine kinase
SANRA	Scale for the Assessment of Narrative Review Articles
TAFA4	TAFA chemokine-like family member 4
TKA	Total knee arthroplasty
TLR4	Toll-like receptor 4
TNF-α	Tumour necrosis factor alpha
TRIPOD + AI	Transparent Reporting of a Multivariable Prediction Model for Individual Prognosis or Diagnosis + Artificial Intelligence
TrkA	Tropomyosin receptor kinase A
TrkB	Tropomyosin receptor kinase B
TRPA1	Transient receptor potential ankyrin 1
TRPV1	Transient receptor potential vanilloid 1
US	United States
